# Comparing allele specific expression and local expression quantitative trait loci and the influence of gene expression on complex trait variation in cattle

**DOI:** 10.1186/s12864-018-5181-0

**Published:** 2018-11-03

**Authors:** Majid Khansefid, Jennie E. Pryce, Sunduimijid Bolormaa, Yizhou Chen, Catriona A. Millen, Amanda J. Chamberlain, Christy J. Vander Jagt, Michael E. Goddard

**Affiliations:** 1 Department of Agriculture and Food Systems, The University of Melbourne, Parkville, VIC Australia; 2 Agriculture Victoria, AgriBio, Centre for AgriBioscience, Bundoora, VIC Australia; 3 La Trobe University, Bundoora, Australia; 4 Elizabeth Macarthur Agricultural Institute, NSW Department of Primary Industries, Menangle, NSW Australia; 5 Agricultural Business Research Institute, The University of New England, Armidale, Australia

**Keywords:** RNA-sequencing, Allele specific expression, ASE analysis, eQTL mapping, Genome-wide association study, Genetic variation of complex traits

## Abstract

**Background:**

The mutations changing the expression level of a gene, or expression quantitative trait loci (eQTL), can be identified by testing the association between genetic variants and gene expression in multiple individuals (eQTL mapping), or by comparing the expression of the alleles in a heterozygous individual (allele specific expression or ASE analysis). The aims of the study were to find and compare ASE and local eQTL in 4 bovine RNA-sequencing (RNA-Seq) datasets, validate them in an independent ASE study and investigate if they are associated with complex trait variation.

**Results:**

We present a novel method for distinguishing between ASE driven by polymorphisms in cis and parent of origin effects. We found that single nucleotide polymorphisms (SNPs) driving ASE are also often local eQTL and therefore presumably cis eQTL. These SNPs often, but not always, affect gene expression in multiple tissues and, when they do, the allele increasing expression is usually the same. However, there were systematic differences between ASE and local eQTL and between tissues and breeds. We also found that SNPs significantly associated with gene expression (*p* < 0.001) were likely to influence some complex traits (*p* < 0.001), which means that some mutations influence variation in complex traits by changing the expression level of genes.

**Conclusion:**

We conclude that ASE detects phenomenon that overlap with local eQTL, but there are also systematic differences between the SNPs discovered by the two methods. Some mutations influencing complex traits are actually eQTL and can be discovered using RNA-Seq including eQTL in the genes *CAST*, *CAPN1*, *LCORL* and *LEPROTL1*.

**Electronic supplementary material:**

The online version of this article (10.1186/s12864-018-5181-0) contains supplementary material, which is available to authorized users.

## Background

Gene expression varies between tissues and individuals and can be measured by counting the number of mRNA copies of the gene [1, 2]. The variation in gene expression between individuals can influence their phenotype [3]. Some of this variation in gene expression is genetic, so understanding gene expression may improve our knowledge of the genetic architecture of complex traits.

In traditional gene expression studies with microarrays, it is not possible to discriminate between eQTL responsible for changing the expression of a gene on the same chromosome (cis eQTL) from mutations influencing the expression of a gene on both chromosomes (trans eQTL) [4]. However, it has become common to regard polymorphisms that are close to the gene whose expression they affect as cis eQTL, although a better name would be local eQTL [5].

**Fig. 1:**
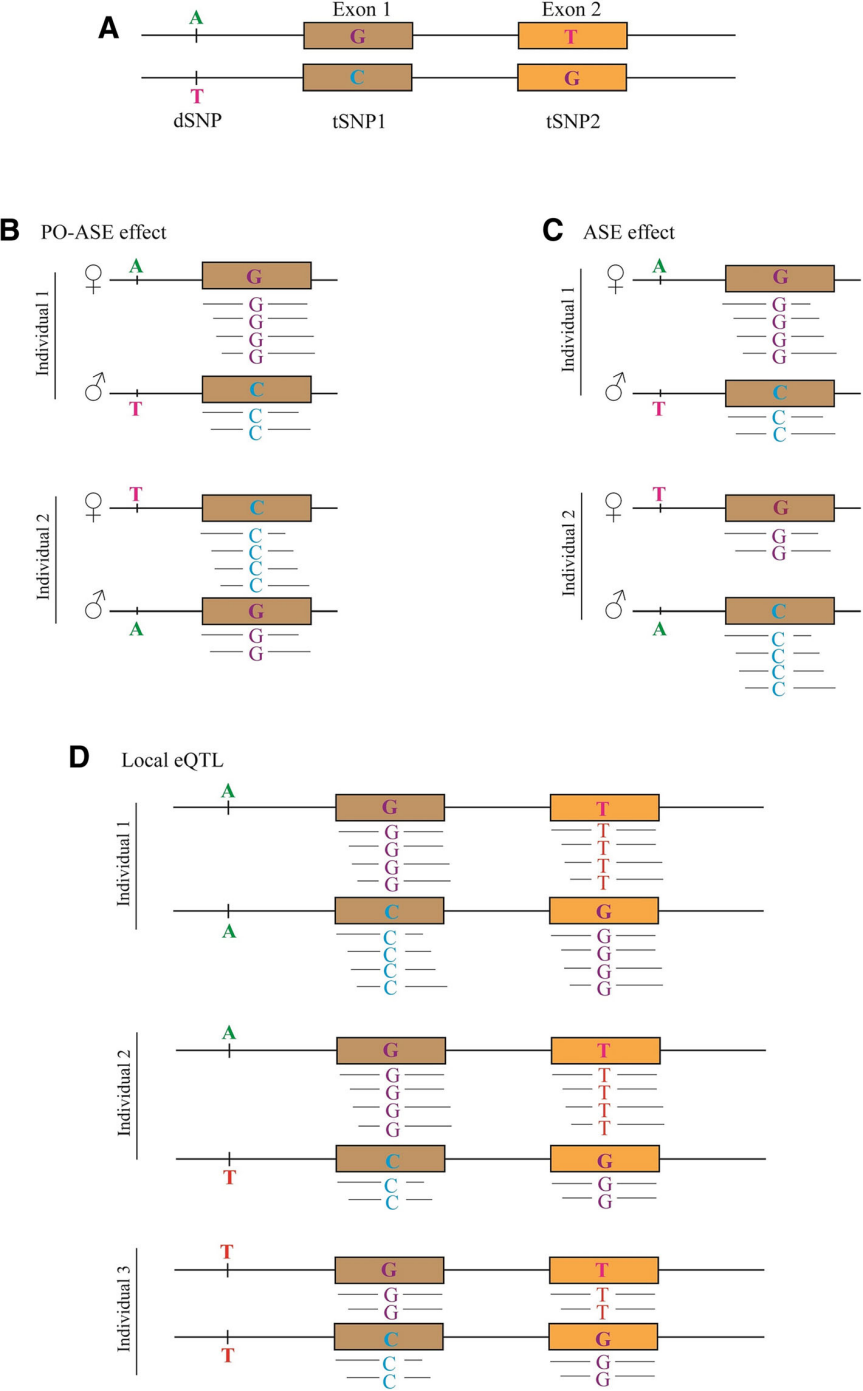
Allele specific expression (ASE), parent-of-origin allele specific expression (PO-ASE) and local expression quantitative trait loci (eQTL) diagram. **a** The gene in the figure has two SNPs in the exons (tSNP_1_ and tSNP_2_), and their expression can be measured using RNA-Seq and a SNP within 50 kb of the gene which is driving expression of the gene (dSNP). **b** In the case of PO-ASE, the allele inherited from the dam (in this example) increases the expression of the tSNP allele which is on the maternal chromosome (♀). **c** When the dSNP has an ASE effect, allele A of the dSNP (in this example) triggers the expression of tSNP allele on the same chromosome. **d** In local eQTL mapping, the expression of dSNP allele is correlated with total exon expression, where the heterozygote individual for dSNP shows medium expression (A/T, individual 2) and the homozygotes show either high (A/A, individual 1) or low (T/T, individual 3) expression

High-throughput RNA sequencing with next generation sequencing technology (RNA-Seq), can be used to measure gene expression and to detect heterozygous sites in the transcript [6]. If there is a polymorphic site in the transcript, then RNA-Seq can also be used to detect allele specific expression (ASE) or allelic bias in which one allele is expressed more highly than the other. To detect ASE, we require a SNP to be in the transcript (tSNP), but the polymorphism causing the difference in expression between the alleles (the driver SNP or dSNP) is likely to be in non-coding, regulatory DNA (Fig. 1a). An individual that is heterozygous for a cis eQTL should show ASE. Therefore, RNA-Seq data can be used for eQTL mapping of global RNA expression and also for ASE analysis, and hence it is possible to distinguish cis eQTL from trans eQTL even among local eQTL [7].

In ASE analysis, the difference in expression of the alleles is a within individual comparison (Fig. 1b and c), while in local eQTL mapping, the association between gene expression level and genotypes at polymorphic loci relies on comparisons between individuals (Fig. 1d). Therefore, the two methods use independent data and combining them should increase power. For the detection of cis eQTL, ASE has an advantage over local eQTL because it relies on comparisons within an individual, so sources of error between individuals, such as environmental or trans eQTL effects, are eliminated [8]. However, this advantage in power is offset by the fact that only RNA reads that include a heterozygous site are useful for ASE, whereas all reads from a gene are useful for local eQTL mapping.

Higher expression of one allele compared with the other can be due to cis eQTL, but it can also be due to parent of origin of allelic expression (PO-ASE, Fig. 1b) or imprinting, where maternal or paternal inheritance of the allele determines the allelic imbalance ratio [9]. Thus, before using ASE to distinguish between cis and trans local eQTL, we need to know whether ASE is largely due to cis eQTL (Fig. 1c), or largely due to other phenomenon such as parent of origin effects. However, to date the reported concordance rates between local eQTL mapping and ASE studies have not been very high due to biological and technical factors [10]. Hasin-Brumshtein et al. (2014) reported only 15–20% of the local eQTL were found as cis eQTL by ASE analysis in mouse adipose tissue [5]. Failure of ASE and local eQTL analyses to agree could be due to insufficient statistical power in one or both analyses, to parent of origin effects or to local trans eQTL. In this paper we examine the extent of systematic differences between local eQTL and ASE.

Distinguishing between ASE due to cis eQTL and parent of origin effects is more complex but more informative in outbred individuals than in crosses of inbred strains. Here we introduce a novel statistical analysis to distinguish between these two causes of allele specific expression. Our novel method can be used to explain some of the discrepancy between the results from ASE analyses and local eQTL mapping.

Finding the biological background of how mutations cause variation in a trait is very useful to confirm the causality of the statistically significant association between mutations and phenotypic records. However, many of the polymorphisms associated with complex traits (quantitative trait loci or QTL), found by genome wide association (GWA) studies, are non-coding variants [8]. Therefore, it seems reasonable to assume that at least some of the causal variants that affect complex traits, do so by regulating the expression of genes. If this is true, we expect that some QTL are actually eQTL. However, only a few QTL have been shown to be eQTL (e.g. in humans the SNP which is located in an enhancer of *Nitric Oxide Synthase 1 Adaptor Protein* gene affects cardiac function by increasing the expression of *NOS1AP*) [3]. Here we examine the overlap between QTL for dairy and beef traits and eQTL.

The gene expression profile and imbalance can be different across tissues and breeds. Therefore, in our study, RNA-Seq of 4 datasets (45 Angus bull muscle samples, 37 Angus bull liver samples and 20 Holstein cow white blood cell (WBC) and liver samples) and corresponding phased genotypes were used to find local eQTL and measure ASE and PO-ASE within 50 kb of the genes expressed in each tissue. We compared the local eQTL, ASE and PO-ASE measurement in different datasets to find if the SNP associated with allelic imbalance in ASE studies and gene expression in eQTL mapping are the same and if so, whether the same allele is associated with higher expression in both cases. We compared the cases of ASE and eQTL found in these 4 datasets to the ASE found in 18 tissues from a single Holstein cow [11], to broaden the range of tissues examined. The SNP associated with gene expression in each RNA-Seq dataset (*p* < 0.001) and SNP associated with 21 complex traits and a multi-trait test (*p* < 0.001), detected by GWAS, were compared to test if QTL are also likely to be eQTL.

The aims of the study were to find and compare ASE and local eQTL, validate them in an independent ASE study and investigate if they are associated with complex trait variation.

## Results

### RNA-Seq quality control

About 80% of the RNA-Seq data passed the quality check (QC) filters. The summary of raw reads, reads passing QC, aligned paired reads and concordant pair alignment rate (%) of the animals in each RNA-Seq dataset, is shown in Table 1. The detailed QC and alignment results are available in Additional file 1: Table S1. On average, approximately 90% of the reads that passed QC were mapped to the reference genomes and the concordant pair rates was about 85%. Concordant read pairs percentage is a good indicator of appropriate alignment of reads to the reference map which shows both pairs mapped to the same strand and in correct orientation and acceptable distance according to the library size.

**Table 1:** Average (and standard deviation) of raw reads, reads passing QC, reads aligned and concordant pair alignment rate in each RNA-Seq dataset

		Raw reads		QC passed reads (paired)		Aligned reads (paired)		Concordant pair alignment rate (%)	
RNA-Seq data	Number of samples	Average	Standard deviation	Average	Standard deviation	Average	Standard deviation	Average	Standard deviation
Angus muscle	45	17,816,260	4,293,439	6,337,427	1,840,633	5,769,907	1,677,758	90.0%	1.4%
Angus liver	37	13,616,027	7,751,116	5,437,947	3,065,337	4,478,339	2,720,511	79.7%	8.4%
Holstein liver	20	35,145,555	7,060,354	14,869,140	3,328,298	13,794,312	2,960,745	91.8%	1.0%
Holstein WBC	20	40,462,263	6,419,677	16,355,951	2,808,778	14,786,745	2,465,978	89.4%	1.1%

The average (and standard deviation) of raw reads, QC passed and aligned paired reads and concordant pair alignment rate (%) of the animals in each RNA-Seq dataset

### ASE and PO-ASE

ASE, in which the ratio of the two alleles in the RNA transcript differs from 1:1, can only be detected if an animal is heterozygous for a SNP in the RNA transcript (i.e. a tSNP). Our statistical analysis distinguishes between two possible explanations. If the paternal allele was always over-expressed (or under-expressed), we interpret this as a parent of origin effect (PO-ASE). Alternatively, if the overexpressed tSNP allele is consistently on the same chromosome as an allele of a nearby SNP (a dSNP), we interpret this as a possible cis eQTL and refer to this phenomenon simply as ASE in the rest of the paper. These two alternatives cannot be distinguished if all heterozygous animals receive the same tSNP allele from their sire. Therefore, we only analyse cases in which at least one animal received the reference allele from its sire and one animal received the reference allele from its dam. An analysis was performed for each combination of a SNP in the transcript (tSNP) and another SNP (dSNP) within 50 kb which could be driving the expression of the gene containing the tSNP. Therefore, a dSNP may be tested for association with ASE more than once if it was within 50 kb of more than one tSNP in the same or different genes. Additional file 2: Table S2 shows the number of tSNP, the number used in analyses of ASE, the number of tests performed and the number of unique dSNP tested. For instance, in the Angus muscle dataset, there were 12,021 tSNP of which 9644 met the requirement to have at least two heterozygous animals who inherited opposite alleles from their sire. In this dataset there were 1,104,748 dSNP tested in 3,749,255 combinations of dSNP and tSNP.

We used a statistical model which fits the parent of origin effect and the cis effect simultaneously and tests the significance of each. The number of dSNP-tSNP pairs tested and significant (*p* < 0.0001) are shown in Table 2. For example, in the Angus muscle dataset, of the 3,749,255 tests performed, 11,717 were significant (*p* < 0.0001) for ASE (compared with 375 expected by chance, giving a FDR of 3%) and 6748 were significant for PO-ASE (FDR = 6%). When all 4 datasets are considered, allelic imbalance at the tSNP is more often associated with the alleles at the dSNP (ASE) than with the parent of origin of the tSNP (PO-ASE) (Table 2).

**Table 2:** Number of SNPs (and genes) tested and number of significant associations (*p* < 0.0001) in ASE, PO-ASE and eQTL analyses

Analysis	Definition	Angus		Holstein		Meta-analysis
		Muscle	Liver	Liver	WBC	
ASE	No. tests	3,749,255 (3022)	3,766,137 (2903)	8,639,073 (5803)	11,419,065 (6873)	20,248,509 (9155)
	No. Sig	11,717 (261)	8250 (79)	5926 (149)	11,299 (194)	1,528,756 (7357)
	FDR	3%	5%	15%	10%	0.1%
PO-ASE	No. Sig.	6748 (67)	594 (18)	1048 (56)	1739 (69)	1,199,496 (6255)
	FDR	6%	63%	82%	66%	0.2%
eQTL	No. tests	8,364,720 (12,278)	7,326,437 (12,233)	9,866,261 (14,373)	9,641,386 (14,175)	13,373,638 (16,038)
	No. Sig.	45,310 (1796)	32,230 (850)	27,107 (579)	22,974 (620)	151,636 (4051)
	FDR	2%	2%	4%	4%	1%
Meta-analysis	No. tests	10,855,138 (12,283)	9,955,525 (12,240)	15,886,606 (14,379)	17,836,290 (14,179)	28,807,218 (16,043)
	No. Sig.	78,539 (1997)	75,667 (950)	84,135 (776)	63,589 (855)	1,377,335 (8317)
	FDR	1%	1%	2%	3%	0.2%

The number of dSNP-tSNP pairs tested and significant (*p* < 0.0001) in ASE and PO-ASE analyses and the number of gene-SNP combinations tested and significant (*p* < 0.0001) in local eQTL mapping are shown in the table. The number SNPs tested or significant in the analyses are associated with fewer numbers of genes which are shown in parenthesis. Generally, combining the results in meta-analyses within and across RNA-Seq datasets reduce the FDRs

To test the validity of the PO-ASE effects, we found 15 genes containing SNPs with significant PO-ASE effects which were previously reported as imprinted genes in human, mouse or cattle according to the GeneImprint database (http://www.geneimprint.com/site/home) and genes reported by Chamberlain et al. (2015) as showing ASE (Table 3) [11]. In 10 out of 15 cases the allele that was over-expressed was the same as previously reported in human, mouse or bovine studies but in some genes the imprinted allele in cattle was different to that in mice or human (*RB1*, *MKRN3* and *Dhcr7*).

**Table 3:** Imprinted genes found in PO-ASE analyses (*p* < 0.01) that have previously been reported in humans, mice and cattle

Gene Name	Bovine Ensemble ID	Expressed allele (Human)^a^	Expressed allele (Mouse)^a^	Expressed allele (Bovine)^a^	Expressed Allele (Bovine)^b^	PO-ASE More expressed allele	RNA-Seq dataset	No. tSNP	No. dSNP
*NAA60*	ENSBTAG00000004875	Maternal	Not reported	Not reported	Not reported	Maternal	Holstein blood	1	1
*RB1*	ENSBTAG00000006640	Maternal	Not imprinted	Not reported	Paternal	Paternal	Holstein blood	1	14
*AIM1*	ENSBTAG00000017527	Paternal	Not reported	Not reported	Not reported	Paternal	Holstein blood	1	9
*PLAGL1*	ENSBTAG00000026523	Paternal	Paternal	Paternal	Not reported	Paternal	Holstein blood	1	3
*DGCR6L*	ENSBTAG00000047299	Random	Not reported	Not reported	Not reported	Paternal	Angus muscle	1	3
*MKRN3*	ENSBTAG00000008306	Paternal	Paternal	Not reported	Not reported	Maternal	Angus liver	1	7
*Gatm*	ENSBTAG00000005586	Not reported	Maternal	Not reported	Not reported	Maternal	Holstein liver	1	13
*Gnai3*	ENSBTAG00000013016	Not reported	Predicted maternal	Not reported	Not reported	Maternal	Holstein blood	1	65
*Dhcr7*	ENSBTAG00000016465	Not reported	Maternal	Not reported	Not reported	Paternal	Holstein blood	1	4
*Tssc4*	ENSBTAG00000047793	Not reported	Maternal	Maternal	Not reported	Maternal	Holstein blood	1	110
*Nap1l4*	ENSBTAG00000022160	Not reported	Maternal	Not reported	Not reported	Maternal	Holstein blood	2	4
*Dcn*	ENSBTAG00000003505	Not reported	Maternal	Not reported	Not reported	Maternal	Angus muscle	2	240
*Igf2*	ENSBTAG00000013066	Not reported	Paternal	Paternal	Paternal	Paternal	Angus muscle	2	5
*Igf2r*	ENSBTAG00000002402	Not reported	Maternal	Maternal	Maternal	Maternal	Holstein liver	3	31
*Impact*	ENSBTAG00000003035	Not reported	Paternal	Not reported	Paternal	Maternal	Holstein blood	1	3

^a^Based on GeneImprint database (http://www.geneimprint.com/site/home)

^b^Based on Chamberlain et al., 2015 [11]

The imprinted genes discovered with PO-ASE effects at *p* < 0.01 and their effects as reported in human, mouse and/or cattle. Most of the imprinted genes with PO-ASE were discovered in Holstein blood RNA-Seq dataset. In *PLAGL1* and *Igf2* the paternal and in *Igf2r*the maternal alleles were expressed according to human, mouse and cattle studies

### Local eQTL discovery

Genes expressed (i.e. RNA-Seq reads that aligned to them) in more than 25% of animals in each dataset were used for eQTL mapping. The expression of each of these genes was tested for association with all SNPs within 50 kb of the gene. The number of genes expressed, number of genes used in eQTL mapping, the number of SNPs and the number of gene-SNP combinations tested for association is shown in Additional file 2: Table S3. The number of significant associations (*p* < 0.0001) between a SNP and gene expression are shown in Table 2. The number of eQTL found by local eQTL mapping was greater than the number found by ASE. Possible reasons for this include more association tests, use of gene counts instead of allele counts, use of all animals instead of heterozygotes, and the possibility that some local eQTL are trans not cis eQTL.

### ASE and local eQTL mapping comparison within and across RNA-Seq datasets

A SNP that affects the expression of a nearby gene might have an effect in only one tissue or multiple tissues. For instance, there were 4,152,056 dSNP-tSNP tests which were performed in both Holstein WBC and Holstein liver. Of these 18,943 were significant in WBC, 10,989 were significant in liver and 1375 were significant in both datasets (*p* < 0.001) which is 27.4 times more than expected if the overlap between the datasets was merely due to chance (the fold enrichment). Of these 1375 associations, the same allele of the dSNP was associated with increasing expression in 100% of cases (Additional file 2: Table S4). Table 4 gives the fold enrichment and the percentage of cases where the effect of an allele is in the same direction in both analyses, for comparisons among the 8 analyses (ASE and local eQTL for 4 datasets). Additional file 2: Table S4, S5, S6 and S7 give more details of ASE, PO-ASE, eQTL and ASE-eQTL comparisons, respectively. The comparisons for PO-ASE in different datasets (Additional file 2: Table S5) shows there are very limited number of significant SNPs shared between datasets and with less agreement in direction of the expression than for ASE.

**Table 4:** Fold enrichment and the percentage of cases where the effect of an allele is in the same direction across different analyses and tissues

Analysis	Tissue	ASE				eQTL			
		Angus muscle	Angus liver	Holstein liver	Holstein WBC	Angus muscle	Angus liver	Holstein liver	Holstein WBC
ASE	Angus muscle		34.82	8.78	3.09	5.89	4.02	9.75	2.00
	Angus liver	100%		11.79	0.09	1.70	11.94	5.34	1.79
	Holstein liver	100%	100%		27.43	1.89	7.80	12.02	0.87
	Holstein WBC	100%	100%	100%		3.72	2.10	8.49	12.23
eQTL	Angus muscle	98%	92%	62%	82%		12.28	7.35	10.84
	Angus liver	100%	98%	99%	61%	94%		20.35	6.00
	Holstein liver	100%	95%	97%	64%	97%	99%		20.36
	Holstein WBC	91%	100%	62%	87%	96%	99%	100%	

The fold enrichment (upper triangle) and the percentage of cases where the effect of an allele is in the same direction (lower triangle) in paired comparisons among ASE and eQTL analyses show that the overlap between significant SNPs (*p* < 0.001) is more than expected by chance and in majority of cases the same allele is associated with higher tSNP count or gene expression

The power to detect ASE in different datasets was not the same because of differences in RNA-Seq read depth and number of samples. So, even if all SNPs that affect expression in WBC also affect expression in liver, we do not expect all tests to be significant in both datasets due to lack of power. However, the lack of power affects all comparisons in Table 4 and so by comparing the fold enrichment in different comparisons we can determine which factors have a systematic effect on the overlap between SNP associated with expression in each analysis. This shows that 3 factors – type of analysis (local eQTL vs ASE), dataset and tissue – have systematic effects on the fold enrichment. For instance, when the overlap between Angus muscle and Angus liver is assessed from ASE, the fold enrichment is 34.8 and from local eQTL it is 12.3, but when one tissue is analysed with ASE and one with local eQTL, the fold enrichment is 4.0 and 1.7 (Table 4). The average for all comparisons across datasets but within method of ASE and eQTL analyses are 14.33 and 12.87, respectively and between methods of analysis is 5.72. This shows that while ASE and local eQTL both detect the same effects in some cases, they detect systematically different effects as well. It appears that only approximately half the cases of ASE and local eQTL are in common. However, in the cases where both ASE and local eQTL are significant, it is usually the same allele of the dSNP that drives over-expression supporting the conclusion that both methods are detecting cis eQTL.

A similar comparison can be done for the difference between datasets. The average fold enrichment when ASE and local eQTL are compared in the same dataset is 10.52 whereas when they are compared in different datasets it is 4.12 (Table 4). Different RNA extraction protocols were used for different tissues, so the differences between datasets could be as a result of this, in addition to differences in breed and physiological status of the animals and tissue. However, gene expression in liver was measured for both Holsteins and Angus. The fold enrichment in ASE, eQTL and ASE-eQTL analyses when 2 liver analyses are compared are 11.79, 20.35 and 6.57 respectively, compared with 3.99, 8.06 and 6.68 when two analyses of different tissues from Angus and Holstein are compared, showing that some eQTL and ASE is specific to a tissue.

The percentage of SNPs where the effect is in the same direction in both analyses (Table 4) shows a similar pattern to the fold enrichment. That is, it is very high when ASE is used for both analyses or when both analyses are for local eQTL but lower when an ASE analysis is compared with a local eQTL analysis. This reinforces the conclusion that ASE and local eQTL find different phenomena in some cases.

Comparisons of ASE in different datasets in Additional file 2: Table S4 use the same dSNP-tSNP pair in both datasets. In parentheses in Table 4 are the fold enrichments and percentage same direction when the same dSNP but not necessarily the same tSNP within each gene is used. In this case the agreement between analyses is reduced. Different tSNPs within a gene are likely to be in different exons, so this reduction in agreement could be due to the effect of the SNP on ASE being exon or transcript specific. The analyses of local eQTL use the transcript count for the whole gene so one reason that ASE and local eQTL analyses differ is that ASE analyses are exon specific and local eQTL analyses are not.

### Combining ASE and eQTL results

The results of meta analyses are shown in Table 2. Generally, combining results in meta-analyses decreased the FDR compared to individual ASE or eQTL analyses. For instance, the FDR for ASE and local eQTL in Angus muscle was 3% and 2% respectively (*p* < 0.0001) which decreased to 1% when combined.

### Validating ASE and eQTL mapping in an independent dataset

To increase the range of tissues considered, we compared our ASE and eQTL analysis with ASE measurements in 18 tissues from a Holstein cow [11]. Detailed results are presented in Additional file 2: Table S8 and S9. In a single cow we do not expect to find all cis eQTL because the cow has to be heterozygous for a tSNP and for the causal variant. Also, we cannot distinguish between possible dSNP because all heterozygous SNPs would give the same results. Therefore, we considered the tSNP as if it was also the dSNP. Despite these limitations we found significant overlap between the ASE and local eQTL found in this study and those found by Chamberlain et al. (2015). On average there were about twice as many SNPs than expected by chance that showed ASE in one of our 4 datasets and one of the 18 tissues of the Holstein cow and, of these, 83% had the same allele at the tSNP overexpressed. For local eQTL the overlap was less than 1.7 times the expected number significant in both experiments and 68% in the same direction. The results for WBC, liver and muscle from the Holstein cow had the highest agreement with our results from the same tissue. Our results from Holstein liver samples had better agreement than our Angus liver samples with the Holstein cow liver results. Hence the results from this single cow confirm the effects of method of analysis, tissue and breed that we found within our own results. Note that breed here is confounded with sex and physiological state. However, there was some overlap between our results and all tissues from the Holstein cow showing that some eQTL at least affect more than one tissue.

### GWAS

GWAS using 800KSNP chip genotypes were performed for 20 traits listed in Additional file 2: Table S10. A multiple trait test, combining these 20 traits was also performed. For residual feed intake, the GWA study was performed on imputed genome sequence data. The number of SNP significantly (*p* < 0.001) associated with these traits is shown in Additional file 2: Table S11 (more detailed results are available in Additional file 3: Table S12 and Additional file 4: Table S13).

### Comparing QTL and eQTL

We used 3 different statistical tests to assess the overlap between QTL found in 21 GWAS (20 complex traits and 1 multi-trait with 800 K SNP chip) and eQTL found in 4 RNA-Seq datasets. Firstly, we calculated a chi-square statistic based on the overlap across the whole genome between QTL for a trait and eQTL and used a permutation test to judge statistical significance. A summary of the results is in Table 5 and more detailed results in Additional file 5: Table S14. The overlap between trait QTL and eQTL is more than expected by chance. The tenderness of the meat (MQLDPF) and the multi-trait test show the most consistent overlap with the eQTL results.

**Table 5:** Traits with a significant overlap between eQTL and QTL (within and across RNA-Seq datasets analyses)

Test	Holstein WBC	Holstein Liver	Angus liver	Angus muscle	All Tissues (meta-analysis)	All Tissues (pooled analysis)
ASE	MQLDPF^*^, Multi-Trait^*^	FI_NFI, MQLDPF^*^, Multi-Trait	E_IGF^*^, MQLDPF^*^, PW_HIP	MQLDPF^*^, Multi-Trait	PW_IGF, X_LWT^*^, Multi-Trait^*^	FI_NFI, MQLDPF^*^, PW_HIP, Multi-Trait^*^
PO-ASE	E_IGF^*^, FI_NFI^*^, PW_LWT^*^		FI_NFI	PW_LWT	PW_HIP, E_HIP	FI_NFI^*^, PW_LWT^*^
eQTL	CRBY^*^, MQLDPF, Multi-Trait	MQLDPF^*^		FI_NFI, MQLDPF^*^, Multi-Trait^*^	MQLDPF^*^	MQLDPF, Multi-Trait
ASE + eQTL (meta-analysis)	CRBY^*^, MQLDPF^*^, Multi-Trait^*^	FI_NFI, MQLDPF^*^	E_IGF^*^, MQLDPF	FI_NFI, MQLDPF, Multi-Trait	CMARB^*^, CRBY, M_SEMA, X_LWT	MQLDPF^*^, Multi-Trait^*^
ASE + eQTL (pooled analysis)	CRBY^*^, MQLDPF^*^, Multi-Trait^*^	MQLDPF^*^, PW_LWT^*^	E_IGF^*^, MQLDPF^*^	FI_NFI, MQLDPF, Multi-Trait		

The traits in which QTL are significantly more likely to be eQTL according to chi-squared tests when comparing GWAS and gene expression results within RNA-Seq datasets analyses (*p* < 0.05). ^*^The approximate correlation between QTL and eQTL for the traits annotated with stars were significant as well (*p* < 0.05)

*CRBY* Carcass retail beef yield; *E_IGF* Blood concentration of Insulin-like Growth Factor I (IGF-I) measured at feedlot entry; *FI_NFI* Net feed intake; *MQLDPF* Tenderness; *Multi-Trait* Multi trait GWAS test; *PW_HIP* Hip height measured post weaning; *PW_LWT* Live weight measured post weaning

Secondly, the test for overlap between eQTL and ASE with trait QTL was also carried out for each gene individually. Summed across all traits and RNA-Seq datasets 9962 tests contained at least one QTL (*p* < 0.001) and one eQTL (*p* < 0.001). The chi-squared tests showed that in 3581 of these tests there was a significant association (*p* < 0.05) such that the SNPs that were associated with the trait were also associated with gene expression (The list of genes and chi-squared tests details are provided in Additional file 6: Table S15).

In a third method, the within gene tests described above were combined into a single test for each trait using a linear model. This showed significant associations between GWAS and gene expression results (*p* < 0.05) for some traits (summarised in Table 5 and detailed results are shown in Additional file 5: Table S14).

### Example of QTL that may be eQTL

**Fig. 2:**
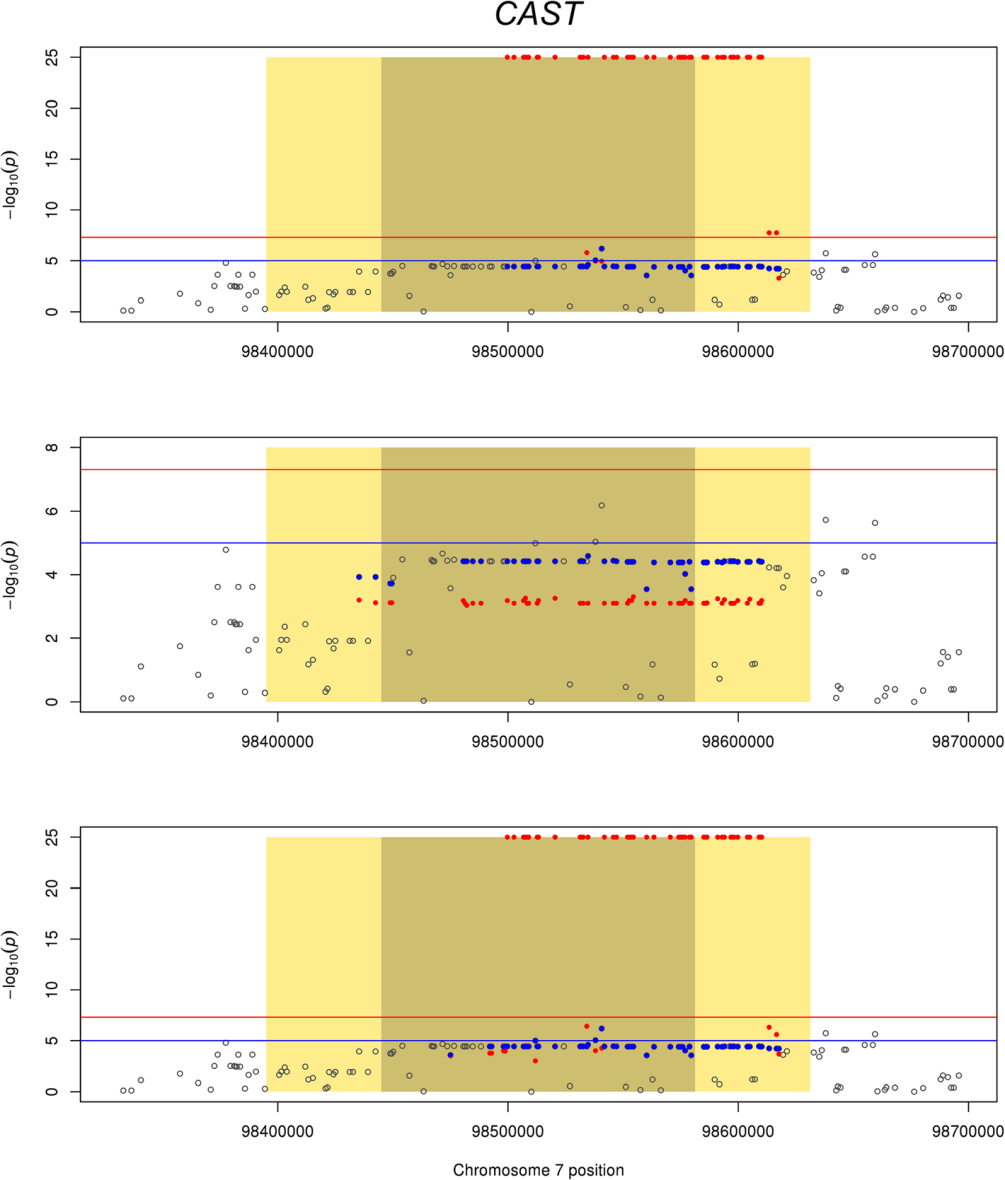
GWAS results for tenderness (MQLDPF) and gene expression results, using RNA-Seq meta-analyses of all datasets, within 50 kb of calpastatin gene (*CAST*). The Manhattan plots show the GWAS results for MQLDPF in grey. The common GWAS suggestive line at *p* < 10^− 5^ and the genome-wide threshold line at *p* < 5 × 10^− 8^ are shown in blue and red, respectively. The dark shaded box indicates the location of *CAST* and light shaded boxes show 50 kb upstream and downstream of the gene. For ease of comparison, the common SNPs from both the GWAS and the gene expression study are plotted in each graph. The SNPs within 50 kb of calpastatin gene significantly associated with MQLDPF (*p* < 0.001) are in blue. The SNP significantly associated with expression of the gene (*p* < 0.001) as detected by ASE (top), local eQTL mapping (middle) and meta-analysis of ASE and local eQTL (bottom) are in red. Where the same dSNP was tested for multiple tSNP, the lowest *p-value* for each dSNP is shown in the graphs. Although ASE and local eQTL mapping are different measurements they show similar results

**Fig. 3:**
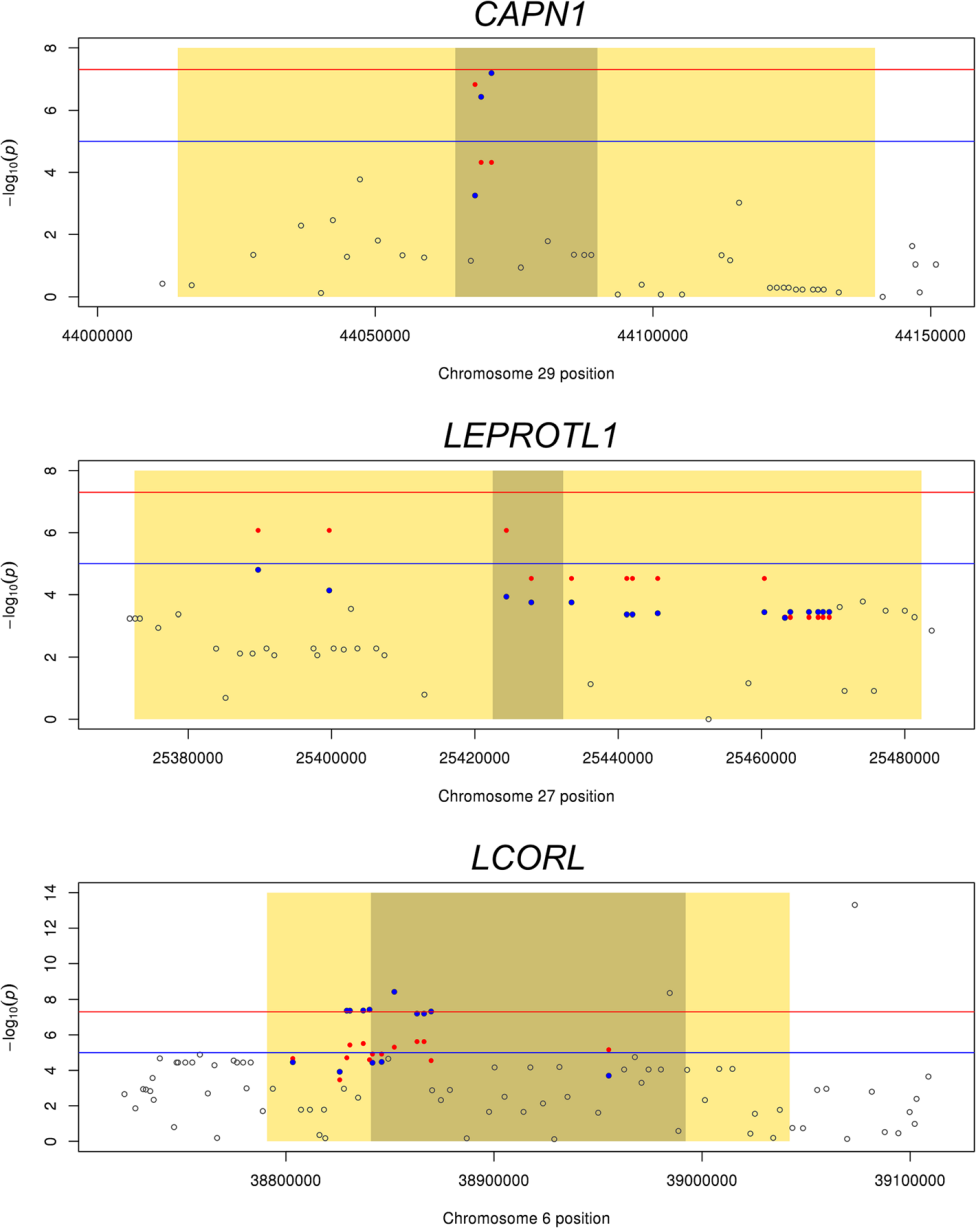
The overlap between QTL and gene expression within 50 kb of calpain (*CAPN1*), leptin receptor transcript-like 1 gene (*LEPROTL1*) and ligand dependent nuclear receptor corepressor-like (*LCORL*) genes. The Manhattan plots show the GWAS results for MQLDPF (top), PW_HIP (middle) and the multi-trait test (bottom) in grey. The common GWAS suggestive line at 10^− 5^ and the genome-wide threshold line at 5 × 10^− 5^ are shown in blue and red, respectively. The dark shaded boxes and light shaded boxes indicate the location and 50 kb upstream and downstream of the genes. For ease of comparison, the common SNPs from both the GWAS and the gene expression study are plotted in each graph. The SNPs within 50 kb of *CAPN1*, *LEPROTL1* and *LCORL* genes, tested in gene expression analyses and significant GWAS (*p* < 0.001) are in blue. The SNPs significantly influenced expression of *CAPN1* and *LEPROTL1* in ASE measurement, and *LCORL* in combined ASE and eQTL meta-analysis of all RNA-Seq datasets (*p* < 0.001) are shown in red

Significant GWAS associations for tenderness (MQLDPF) and significant gene expression results, using meta-analysis of RNA-Seq datasets, were found near the *calpastatin* gene (Fig. 2) which has previously been reported in several papers as a candidate gene for meat quality (e.g. Bolormaa et al., 2013) [12]. The majority of QTL significantly associated with MQLDPF and located within 50 kb of *calpastatin* are also associated with the expression of the gene. Fig 3 shows 3 other genes (*CAPN1*, *LEPROTL1*, *LCORL*) where there are SNPs that are significantly associated with a trait and with expression of a nearby gene.

## Discussion

A sequence variant that affects the expression of a gene on the same chromosome (a cis eQTL) should be detectable both as a local eQTL and by ASE. However, both local eQTL and ASE could be due to other causes besides a cis eQTL, for example local eQTL could be trans eQTL [5]. ASE could be due to parent of origin effects (i.e. imprinting) or nonsense mediated decay [5, 11]. Our statistical analysis attempts to distinguish cis eQTL from parent of origin as a cause of ASE by determining whether the direction of ASE is consistent with the parent of origin of the over-expressed allele or with the allele on the same chromosome at a nearby SNP (a dSNP). This analysis finds that PO-ASE is less common and has higher FDR than ASE due to the cis effect of a nearby SNP. The PO-ASE effects were also less often confirmed in multiple datasets. However, our analysis did find PO-ASE for several genes where imprinting has been reported. To avoid being misled by errors in the genome sequence of our cattle, we excluded complete mono allelic expression, and therefore would not find cases of complete imprinting where only one parental allele is expressed. Therefore, our PO-ASE tests are detecting “partial” imprinting. There are also some reports that imprinting can be tissue specific [13], which could explain why PO-ASE effects were not always confirmed in multiple datasets. However, the agreement of PO-ASE found in Holstein and Angus liver samples was also low perhaps because we had limited power to detect partial imprinting with only a small allelic imbalance.

Our analysis of ASE fits the effects of parent of origin and of a SNP in cis with the over-expressed allele jointly and therefore we can test for significance the cis effect of SNPs on gene expression, called here simply ASE. There are large numbers of SNPs associated with the expression of a nearby gene (local eQTL), or with ASE at that gene (Table 2). The SNPs associated with gene expression overlap significantly between ASE and local eQTL analyses and between datasets on different animals and different tissues and, among the SNPs that are significant in two different analyses, the same allele is associated with increasing gene expression most of the time (Table 4). These results support the conclusion that many of the SNPs associated with local eQTL and with ASE are cis eQTL.

Despite the significant overlap between local eQTL and ASE, only a small proportion of SNPs that are significant in one analysis are significant in the other. This could be due to lack of power in one or both analyses or systematic differences between ASE and local eQTL. When ASE in two different datasets is compared, there are on average 14.33 times more SNPs that are significant in both datasets than expected by chance (Table 4). Similarly, when local eQTL are compared in 2 datasets there are 12.87 times more significant SNPs than expected by chance (Table 4). However, when ASE in one dataset is compared to local eQTL in another, the fold enrichment is only 5.72 (Table 4). This indicates that ASE detects phenomenon that overlap with local eQTL, but have some systematic differences. One reason for these differences is that the local eQTL analysis uses all RNA reads from the gene whereas the ASE analysis uses only those containing the tSNP. When ASE in different datasets is compared using any tSNP within the gene, instead of the same tSNP in both datasets, the fold enrichment falls to 4.81 (Additional file 2: Table S4). Thus, some of the cis eQTL detected by ASE are tSNP specific probably because they are exon and splice variant specific [14]. Other reasons for systematic differences between ASE and local eQTL may include local eQTL being trans eQTL, nonsense mediated decay, and feedback mechanisms limiting gene expression. If one allele is more highly expressed than the other, feedback might limit expression of both alleles so that the total gene expression is much the same regardless of SNP genotype [8]. This would leave a significant case of ASE but no local eQTL.

Although there is some overlap between eQTL in different datasets, there are also systematic differences between datasets, breeds and tissues. The comparisons between ASE and local eQTL mapping showed within an RNA-Seq dataset (Additional file 2: Table S7), the SNPs found by ASE were more likely to be found in local eQTL mapping than expected by chance. The average fold enrichment for ASE and local eQTL in the same dataset was more than when comparing ASE in one dataset with local eQTL in another dataset, indicating that although some eQTL operate in both datasets, there are some dataset specific (tissue specific) eQTL. These results could be interpreted to mean that approximately half the cis eQTL in one tissue also operate in a second tissue which is the same conclusion as the GTex paper reached by different means [1]. Our results also show that when a cis eQTL operates in multiple tissues, it is nearly always the same allele that increases expression.

The systematic differences between datasets are due partly to differences in the tissue used, as shown by the similarities in local eQTL mapping and ASE between Holstein and Angus liver samples (Table 4). The ASE and local eQTL results were also more similar when comparing two Holstein or two Angus datasets than when comparing one Angus and one Holstein dataset (Table 4). The effect of breed might be due to differences between breeds in linkage disequilibrium, but could also be due to differences in gender, environment or physiological state between the Holsteins and Angus cattle we sampled.

These conclusions of the effect of tissue and breed are supported by comparing our results with ASE in 18 tissues of a Holstein cow. In these results, the percentage of SNPs showing the same direction of effect is higher when the tissues are the same and when the breed is Holstein in both cases. So, it seems that ASE and local eQTL are partially tissue specific which agrees with the findings of Chamberlain et al. (2015) [11]. However, there was overlap between all of our 4 datasets and many of the 18 tissues suggesting that some cis eQTL affect expression in many tissues. This is consistent with the GTex paper [1] that found the most cis eQTL either affected all 9 tissues or only one tissue.

There were significant (*p* < 0.05) overlaps between QTL influencing some complex traits and ASE or local eQTL (Table 5). Tenderness of the meat (MQLDPF) was associated with many SNPs that were also significantly associated with gene expression in multiple analyses and datasets. Fig 2 gives an example of a gene containing SNPs that significantly affect tenderness and expression of the gene *calpastatin* (*CAST*). The physiological role of *CAST* and the association between SNPs near *CAST* and tenderness are well known [15–17] but these results suggest that the known QTL is in fact an eQTL for *calpastatin* expression. Further examples include the *calpain 1* gene (*CAPN1*), *leptin receptor overlapping transcript-like 1* gene (*LEPROTL1*) and ligand dependent nuclear receptor corepressor-like (*LCORL*) (Fig. 3). *CAPN1* influences meat tenderness [16]. *LEPROTL1* is reported to influence body growth by negatively regulating leptin receptor (*LEPR*) cell surface expression, decreasing response to leptin and decreasing hepatic growth hormone action in mice [18]. *LCORL* has been reported to have an effect on feed intake, gain, meat and carcass traits [19] and its expression in muscle tissue has been associated with average daily feed intake in beef cattle [20]. In humans, there is a QTL for height near *NCAPG* and *LCORL* and it has not been possible to identify the causal gene. The result here suggests that *LCORL*, at least, is likely to affect growth.

There are many QTL for which we did not find a matching eQTL. This could be because the QTL acts through a different mechanism (e.g. a protein coding mutation) or due to the lack of power to find the relevant eQTL in our experiment. In our study gene expression was measured in just 3 tissues (muscle, liver and WBC) and only once. The eQTL in other tissues and their activity in other physiological or developmental states might underlie other QTL. The number of animals and the average transcript read depth in our study were also limiting factors in the power to detect eQTL. Therefore, if we want to find QTL influencing complex traits by controlling the expression of genes, it is important to sample the correct tissue at the appropriate time and to have sufficient sample size and transcript read depth for gene expression analyses.

## Conclusions

Our statistical analysis distinguishes allelic bias due to parent of origin effects from that driven by a cis acting regulatory allele. The parent of origin effects detected in our study include well known imprinted genes (e.g. *igf2r*) but also partial imprinting that is restricted to one dataset, perhaps because it is tissue, or animal, or time dependent.

In our study, many genes show local eQTL and/or ASE. When the same SNP drives both, it is most likely a cis eQTL. Consequently, combining the two types of analysis increases the power to detect cis eQTL. However, there are some systematic differences between local eQTL and ASE caused by exon specific ASE, local trans eQTL, nonsense mediated decay and perhaps other phenomena such as feedback mechanisms.

In the tissues we studied, approximately half cis eQTL are shared between a pair of tissues such as liver and muscle. When they are shared, usually the same allele is over-expressed in both tissues. Moreover, some cis eQTL are shared between Angus bulls and lactating Holstein cows but some are not.

The QTL of complex traits in beef cattle are slightly enriched for cis eQTL. More QTL would be identified as eQTL if there were more animals, tissues, developmental and physiological states and greater sequencing depth. Despite the mentioned limitations in our study, we conclude the QTL affecting beef tenderness and growth are most likely cis eQTL for *calpain 1*, *calpastatin* and *LCORL*.

## Methods

### Animals

Samples from 82 Angus bulls (45 with muscle and 37 with liver samples) from lines of cattle divergently selected for residual feed intake (RFI) were taken from the Agricultural Research Centre, Trangie, NSW, Australia [21] and 20 first lactating Holstein cows (each of them with both WBC and liver samples) were taken from the DEDTR Ellinbank Research Farm, VIC, Australia [22].

For the GWA studies, 20 complex traits were used from 3296 *Bos taurus* cattle, which were a subset of 10,191 *Bos taurus, Bos indicus* and *Bos taurus*×*Bos indicus* crosses used in a multi-trait test [23]. The names and abbreviations of the traits are shown in Additional file 2: Table S10. The phenotypes and genetic parameters estimated using these data are described in full by Bolormaa et al. (2014) [23]. In another GWA study for RFI, 5614 *Bos taurus* cattle described by Khansefid et al. (2014) were used [24].

### Genotypes

The Angus bulls that had RNA-Seq data, also had Illumina BovineSNP50 (liver samples) or Illumina BovineHD SNP genotypes (muscle samples). The low density SNP were imputed to high density (HD) genotypes using BEAGLE [25]. The Holsteins had BovineHD SNP genotypes, described by Pryce et al. (2012) [26]. Additionally, 45 Angus bulls with muscle samples also had whole genome sequence (WGS) data with an average coverage of 6.7 fold [27].

The animals in GWA studies had 729,068 SNP genotypes, genotyped using either HD chip or a lower density SNP chip and then imputed to HD using BEAGLE [25] as described by Bolormaa et al. (2014) [23].

The HD SNP genotypes of animals with RNA-Seq data and 5614 animals used in GWA study for RFI were imputed and phased to WGS of 28,899,038 SNPs using FImpute [28]. The WGS genotypes of 45 Angus bulls were also phased by FImpute. The reference genomes used for the imputation were WGS data in Run 4.0, 1000 bull genomes project [27], consisting of 27 breeds and 1147 sequenced animals, including 138 Angus (Black and Red) and 311 Holstein cattle (288 black and white and 23 red and white).

### RNA-Seq data

We included here the tissue sampling procedures and RNA-Seq protocol as reported by Khansefid et al., 2017 [29] for completeness in this manuscript. All animals were monitored for one week post procedure for complications before being returned to the main herd at their respective farms.

The beef cattle liver tissue sampling procedure was described in detail by Chen et al., 2011 [30]. Briefly, each animal was sedated with intramuscular 2% xylazine (Xylazil-20, Ilium) and complete local anaesthesia was obtained with Adrenaline by under skin infiltration. On the right side, being about halfway down the curvature of the ribs, the cannula was pushed to penetrate the liver and then rotated and advanced to a depth of 3–5 cm to cut out a biopsy. The procedure was carried out by a professional veterinarian and cut sites were consistent among animals. Liver biopsy samples were expelled into 2 ml tubes of RNAlater™ solution (Ambion, Applied Biosystems). The semitendinosus muscle samples were taken from the growing bulls by a purpose built 12 V powered motorised biopsy drill. The hairs were clipped from a 10 cm × 10 cm area around the point of incision (located 15 and 25 cm below the anus, and over the “poverty groove” of the hind leg) and then that area was wiped with gauze soaked in 70% ethanol solution. Local anaesthetic (5 ml Lignocaine®) is infiltrated under the skin, and a single 1 cm incision is made through the skin using a sterile scalpel. The biopsy needle (approx. 5 cm long and 3 mm inside diameter), attached to the drill was then passed into the muscle with a simultaneous vacuum applied to hold the sample in the biopsy needle and then transferred into 2 ml RNAlater™ solution (Ambion, Applied Biosystems). Total RNA was extracted from liver and muscle tissue by TRI Reagent® (Ambion) and Qiagen RNeasy MinElute kit (Qiagen) using a modified protocol. Approximately 30 mg of liver tissue samples (100 mg of muscle tissue), were finely minced and then mixed with 500 μL TRIzol® Reagent (Life Technologies). The mix were immediately homogenized for approximately 45–50 s and incubated at room temperature for 5 min. The resulting lysate was mixed with 100 μL BCP (1-Bromo-3-chloropropane) incubated at room temperature for 10 min, followed by centrifugation at 10000 g at 4 °C for 15 min. The top aqueous layer was transferred to a new microfuge tube and mixed with an equal volume of 75% ethanol. The resulting lysate was then loaded onto the Qiagen RNeasy MinElute column and RNA was purified per protocol with on column DNA digestion. The extracted RNA quality was assessed using an Agilent Bioanalyzer 2100 (Agilent Technologies) to have integrity number (RIN) of more than 7.0. Ten μg of the extracted RNA from the sampled tissues were enriched with Dynabeads® mRNA Purification Kit (Invitrogen). The cDNA molecules were prepared, barcoded with 24 unique adaptors, enriched by PCR with Phusion® High-Fidelity DNA Polymeras (New England Biolabs Ltd), purified with Agencourt AMPure XP (Beckman Coulter) and selected for target size of 200 bp. Libraries were individually barcoded, pooled and run on a HiSeq™ 2000 (Illumina Inc.) in a 101 bp paired-end run.

For the Holsteins, the blood sampling and liver tissue biopsy, RNA extraction and sequencing were described by Chamberlain et al., 2015 [11] and Khansefid et al., 2017 [29]. In brief, approximately 30 ml of whole blood was collected by venipuncture of the coccygeal vein using BD Vacutainer® Blood Collection Tubes and then centrifuged at 2000 g for 15 min. The white blood cells (about 1500 μl for each animal) were separated and stored in 1.2 ml RNAlater® RNA Stabilisation Solution (Ambion, Applied Biosystems). Liver biopsies were collected by restraining cows in a crush and giving them 10 ml of lignocaine hydrochloride 2 % into the subcutaneous, inter-costal and peritoneal tissues at the site of the insertion of the biopsy punch. A small incision was made with a scalpel before a biopsy punch was inserted into the liver to collect approximately 2–3 g of tissue. Following removal of the biopsy punch, betadine cream was placed in the incision site. Cows were given intramuscular antibiotics (Excenel RTU 2 ml/100 kg) and anti-inflammatory drugs (Ketoprofen 2 ml/100 kg) before being released from the crush. Immediately following collection samples were frozen in liquid nitrogen and then stored at − 80 °C. RNA was extracted from WBC using the RiboPure™ Blood Kit (Ambion, Applied Biosystems) and from liver samples using the RiboPure™ Kit (Ambion, Applied Biosystems) according to the manufacturer’s instructions. All samples were assessed to have RIN greater than 8.0. Sequencing libraries were prepared using the TruSeq™ RNA Sample Preparation Kit v2 Set A (Illumina, Inc.) and selected for size of 200 bp. All libraries were uniquely barcoded, pooled and sequenced on a HiSeq™ 2000 (Illumina Inc.) in a 105 bp paired-end run.

After mRNA sequencing, the raw reads were passed through quality control (QC) filters. Reads were trimmed of adaptor sequence and bases with qscore < 15, where 3 consecutive bases had a qscore of less than 15, the rest of sequence was removed. Reads with minimum average qscore < 20 or read length < 50 after trimming were removed and only the paired reads used in alignment [31].

### Alignment of RNA reads

To improve the mapability of reads, 2 customized reference genomes (paternal and maternal) were made for each animal using 28,899,038 phased SNP genotypes as described by Chamberlain et al. (2015) [11]. This strategy reduced bias in the counts towards the alleles represented in the reference genome [32, 33]. For each animal, the RNA-Seq reads were aligned to its maternal and paternal customised reference genomes using TopHat2 [34] with default input parameters and annotation release 75 GFF file (General Feature Format) of bovine genome assembly UMD3.1.

### Abundance of alleles and genes

#### Counting alleles with Samtools mpileup

The SNP genotypes in WGS data (real or imputed) were used to find heterozygote SNPs in each animal and confirm that each heterozygote SNP observed in RNA-Seq reads was not a sequencing error or the result of mismapped reads. However, the maternal and paternal reference genomes contain errors as well, because the DNA sequence depth for the animals with WGS was relatively low and the remaining animals had imputed WGS genotypes. To minimize errors in detecting ASE because of sequencing and imputation errors, heterozygote SNP in RNA-Seq data had to have at least 1 count of each allele, thereby excluding cases of strict mono allelic expression, and a coverage > 10. We used Samtools mpileup [35] command to measure the number of alleles expressed in RNA-Seq data for heterozygote SNPs found in WGS genotypes of each animal. As we mapped the RNA-Seq reads to 2 reference genomes for each animal, we had two counts for each allele. If the counts of alleles from maternal and paternal alignments were different due to mapping bias, the average of the two counts was reported.

#### Counting genes with HTSeq

The total number of reads mapped to each gene in the reference genome was counted using the HTSeq python package [36]. We assumed some reads were not mapped to the reference genome due to the number of mismatches. When the reads could map to either the maternal or paternal reference genome slightly better, we used the superior one as the reference genome to measure the gene abundance.

### Statistical analysis of ASE

To detect ASE, we require a SNP in the transcript (tSNP), however the causative variant responsible for differences in expression may be a SNP close to the gene, referred to here as a driver SNP (dSNP, Fig. 1a). Allelic imbalance can be the result of PO-ASE (Fig. 1b) or ASE (Fig. 1c) [8]. In order to distinguish ASE from PO-ASE in animals heterozygous for the tSNP, we used the log_10_ of the ratio of maternal to paternal allele counts and included the total abundance of alleles (T) as the weight (the reciprocal of the error variance in eq. 1). A separate analysis was performed for each combination of a tSNP and a dSNP within 50 kb of the tSNP.

1 $$ {\boldsymbol{y}}_{\boldsymbol{SNP}}=\mathbf{1}\upalpha +{\boldsymbol{X}}_{\mathbf{1}}{\boldsymbol{b}}_{\mathbf{1}}+\boldsymbol{e} $$

**y**_**SNP**_ is an N × 1 vector, where y_SNP i_ = log_10_(maternal allele count / paternal allele count) for animal *i* at the tSNP and N is the number of heterozygous animals at the tSNP. **1** is an N × 1 vector of 1’s and ɑ is a scalar intercept that indicates a parent of origin effect. **X**_**1**_ is an N × 1 vector coding the genotype of each animal at a dSNP which may be driving the expression of the tSNP such that homozygotes are coded 0, heterozygote with the reference allele (UMD3.1) inherited from the sire is coded − 1 and a heterozygote with reference allele from the dam is coded + 1. b_1_ is a scalar measuring ASE due to the allele carried at the dSNP. **e** is the vector of random residual effects **e**~N(0, T^− 1^**I**σ^2^_e_).

We restricted the data analysed to tSNPs where there were at least two heterozygous animals who had received opposite alleles from their sire so that we could distinguish PO-ASE from other cases of ASE. The variants within 50 kb of tSNP were used as dSNP to see if these variants regulate the expression of the tSNP. So, if the parent of origin of the allele controls the allelic imbalance (Fig. 1a), individuals with − 1, 0 and + 1 genotypes show the same allelic expression frequency and the intercept (ɑ) would be significantly different from 0. On the other hand, if the actual allele of the dSNP regulates the expression (Fig. 1c), homozygote individuals show no allelic imbalance and individuals with − 1 and 1 genotypes have reversed allelic imbalance which influences the slope of the regression line (b_1_).

The number of significant effects of ɑ and b_1_ were calculated (*p* < 0.001). The FDR was calculated with eq. 2 [12].

2 $$ FDR=\frac{P\left(1-\frac{A}{T}\right)}{\left(\frac{A}{T}\right)\left(1-P\right)} $$

where P is the *p*-value of the test, A is the number of SNP that were associated with gene expression at P and T is the total number of SNP tested.

### Local eQTL mapping

In local eQTL mapping, the effect of the dSNP on the overall expression of a gene was measured (Fig. 1d). The genes expressed in more than 25% of animals in each group were used to find eQTL separately. The gene counts were normalized with a weighted trimmed mean (TMM) of the log expression ratios and the library size for each animal was adjusted to have equivalent counts per million (cpm) using edgeR [37]. Normalizing the counts minimizes the compositional difference between the libraries and calculating cpm removes the effect of sequencing depth on the variation of gene counts in different samples. Finally, log_10_ of the counts were used to calculate the association between the abundance of a gene and the dSNPs with ASReml [38] for each group independently using eq. 3.

3 $$ {\boldsymbol{y}}_{\boldsymbol{gene}}=\mathbf{1}\upalpha +{\boldsymbol{X}}_{\mathbf{2}}{b}_2+\boldsymbol{Zu}+\boldsymbol{e} $$

**y**_**gene**_ is an N × 1 vector where y_gene i_ = log_10_(normalized gene count) for animal *i*, **1** is an N × 1 vector of 1’s and ɑ is a scalar intercept. **X**_**2**_ is an N × 1 vector containing the coded genotypes at the dSNP such that x_2i_ = 0, 1 or 2 if animal *i* has 2, 1 or 0 copy of the reference genome (UMD3.1), respectively and b_2_ is a scalar measuring the effect of the dSNP on gene expression. **Z** is an N × M matrix allocating records to animals where M = number of animals in the pedigree and **u** is an M × 1 vector with u_i_ = the genetic effect of animal *i* on gene expression ~ N(0, **A**σ^2^_u_) while pedigree information was used to make the relationship matrix (**A**). **e** is the vector of random residual effects **e**~N(0, **I**σ^2^_e_). The number tests showing significant association between SNP and gene expression (*p* < 0.001) and the corresponding FDR were calculated.

### Comparing ASE, PO-ASE and local eQTL mapping within and across RNA-Seq data

Within each RNA-Seq dataset, the ASE, PO-ASE and local eQTL mapping results were compared to see if the same SNPs were significantly associated with ASE and gene expression in the same or different datasets. Firstly, a 2 × 2 contingency table was constructed by classifying a SNP as significant or not (*p* < 0.0001) in two datasets. Then a chi-squared test was used to test if SNPs were significant in both datasets more often than expected by chance according to the proportion of significant SNPs in each dataset. The degree of overlap was calculated as the observed number of SNPs significant in both datasets divided by the number expected if overlap was due to chance and referred to as ‘fold enrichment’. Secondly, for SNPs significant in both datasets, the proportion in which the allele increasing expression was the same in both datasets was calculated. The ASE, PO-ASE and local eQTL results were compared and fold enrichment was calculated across the 4 RNA-Seq datasets to see if mutations that were detected in one dataset can be found in the other ones. For ASE and PO-ASE comparison for each gene, we compared results based on the same tSNP and same dSNP. However, we also made some comparisons for the same dSNP but any tSNP within that gene. The comparisons involving local eQTL are only matched on dSNP and gene since no tSNP is used in eQTL analysis.

### Validating ASE and local eQTL mapping in a dataset consisting of 18 tissues

The ASE and eQTL mapping results in the 4 RNA-Seq datasets analysed here were compared with ASE measurements in another study of 18 tissues of a lactating Holstein cow [11] to investigate whether the same SNPs were associated with ASE and gene expression in other tissues. To test the significance of the overlap a chi-squared test, similar to that used to compare datasets, was performed and the proportion of SNPs in which the same allele was associated with increased expression was calculated. This comparison was only carried out using the same tSNP and treating it as the driver SNP as well. Furthermore, the average similarity between ASE and eQTL mapping results of Angus and Holstein cattle liver samples in our study with the validation dataset was tested using a paired t-test. We expected to see more similarity between our Holstein RNA-Seq dataset and the validation data because they were the same breed and all were lactating cows.

### Combining ASE and eQTL within and across RNA-Seq data

ASE and local eQTL analysis both detect cis eQTL, therefore combining the results from both analyses should improve the power of detecting variants influencing the expression of genes. So, the ASE and local eQTL mapping results were combined in a meta-analysis within each dataset. In addition, ASE, PO-ASE and eQTL mapping results across all datasets were also combined. Additional file 2: Figure S1 depicts all within and across RNA-Seq datasets analyses.

In the meta-analyses for any of the within or across RNA-Seq datasets, if we had multiple estimates (n) of the effect (U_i_) of a SNP on expression of a gene, we calculated a weighted average of these estimates as follows: Each U_i_ was converted to a signed t-value (=U_i_/s_i_ where s_i_ = standard error of U_i_). Then the *p*-value of this t statistic was calculated (p_i_) using the appropriate degrees of freedom and the equivalent z value for this p was determined by z = Ф^− 1^(p) and Ф is the cumulative standard normal distribution. The z scores were given the same sign as the original effect U_i_ and finally z̅^*^ was calculated with eq. 4 where z̅^*^ is approximately distributed N(0,1).

4 $$ {\overline{z}}^{\ast }=\sqrt{\frac{{\left(\sum \limits_{i=1}^n{z}_i^{\ast}\right)}^2}{n}} $$

In pooled analyses we merged the tests in the combined datasets and kept the original effects of SNPs, so some SNPs could have multiple solutions.

### GWAS

For the GWAS of 20 traits for *Bos taurus* cattle with BovineHD SNP genotypes we used ASReml and the same model as Bolormaa et al. (2014). In addition, the multi-trait GWAS results were obtained from the study of Bolormaa et al. (2014) [23]. The genotypes for 729,068 SNP passed the quality control filters as described by Bolormaa et al. (2013) [12]. However, the number of SNP with minor allele frequency (MAF) greater than 0.005 were not equal for all traits because not all cattle were measured for all traits.

An additional GWA study for RFI in *Bos taurus* cattle was performed on imputed genome sequence (24,041,262 SNPs with MAF > 0.001) with ASReml using the model and cattle described by Khansefid et al. (2014) [24].

### Comparing QTL and eQTL

**Fig. 4:**
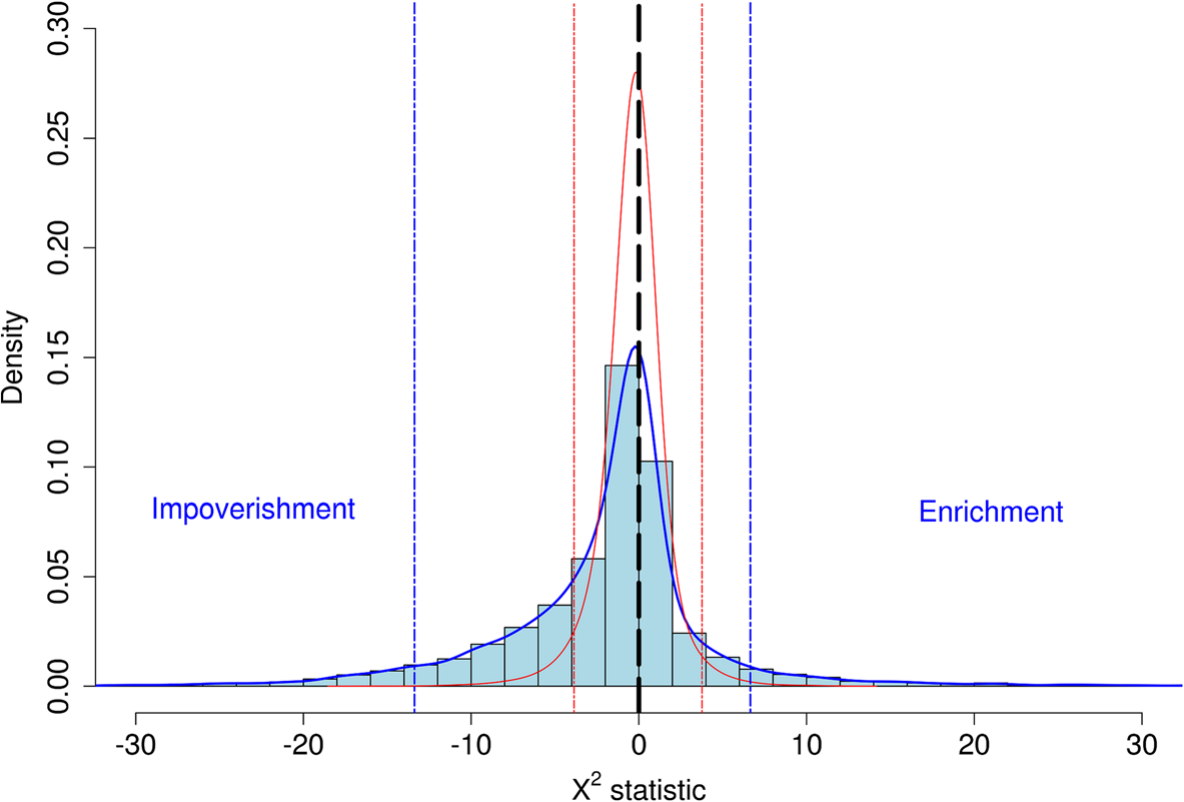
The distribution of the Χ^2^ statistic for the association between QTL and eQTL from a permutation test. This example is from the comparison of eQTL from a meta-analysis of all tissues and the multi-trait GWAS. The enriched and impoverished overlapping QTL and eQTL are shown in positive and negative values, respectively. The theoretical thresholds for a Χ^2^_df = 1_ test is 3.84 (enrichment) and − 3.84 (impoverishment) for significance level *p* < 0.05 as are shown by red dotted lines. The empirical thresholds based on the highest and lowest 5% of values in 10,000 permutation tests, are shown with blue dotted lines. Therefore, in the eQTL meta analysis test, the X^2^ for an enrichment test should be greater than 6.64 for significance level *p* < 0.05

We compared GWAS with expression results to find whether SNP associated with conventional phenotypes (QTL) are also associated with gene expression in the local eQTL or ASE analysis. The hypothesis, that SNP significantly associated with the traits (*p* < 0.001) were more likely to be associated with gene expression (*p* < 0.001) than expected by chance, was tested using a chi-squared test (H_0_: QTL and eQTL are independent). However, the assumption of the chi-squared test is that the SNPs are independent. This is not warranted because nearby SNPs are likely to be in linkage disequilibrium. Consequently, significant SNPs for both traits and gene expression tend to be clumped together on the genome. To overcome the limitation of the chi-squared test 3 other tests were performed:

1) To derive a valid distribution of the Χ^2^_df = 1_ statistic under the null hypothesis, that eQTL and QTL are independent, a permutation test was used. The vector of GWAS results was shifted by 10 to 90% of number of SNPs, and the overlap between the QTLs in the new vector and the eQTL was calculated as a chi-square statistic. This was repeated 10,000 times to generate the distribution under the null hypothesis. Each replicate of the permutation tests can show either more overlap between QTL and eQTL than expected by chance (enrichment) or less overlap than expected by chance (impoverishment), so the replicates were divided into these two groups and separate distributions calculated for the Χ^2^ statistics according to whether the replicate showed enrichment or impoverishment. For example, the X^2^ statistics from the permutation test comparing the meta analysis eQTL (combined ASE and eQTL mapping results in all tissues) with QTL found in a multi-trait GWAS test, is shown in Fig. 4, where the permutations with impoverished overlapping were given negative signs. To find the appropriate thresholds from the 10,000 permutations, the Χ^2^ values for each replicate was calculated and, after changing the sign of impoverished replicates to negative, the Χ^2^ values were sorted from smallest to the largest. The thresholds that separate the top 5% and bottom 5% (i.e. Χ^2^ with rank 500 and 9500, respectively) are marked on Fig. 4 as blue dotted lines and were used to test the experimental X^2^ statistic and declare them as significant or not at *p* < 0.05 for a one-tail test since only enrichment is of interest. In Fig. 4 these thresholds are Χ^2^ = − 13.38 and 6.64 for significance level *p* < 0.05 in the tests for impoverishment and enrichment respectively. In the permutations experiments, the replicates with less than expected overlapping QTL and eQTL (impoverishment) were more frequent than replicates with enrichment because the GWAS results include a few clusters of QTL and shifting the position of the eQTL is likely to place them between clusters causing impoverishment. So, in the majority of permutation experiments, the calculated threshold for a significant impoverishment was a larger value than for significant enrichment. For comparison, the theoretical Χ_(1)_^2^ distribution is shown in red and the thresholds for 5% (− 3.84 and 3.84) for tests with impoverishment and enriched overlapping are annotated in red vertical dotted lines for significance level *P* < 0.05.

2) For each gene individually, SNPs were classified as significant or not for both gene expression and the trait leading to a 2 × 2 contingency table. The significance of the overlap between SNPs significant as QTL and as eQTL was tested by a Χ^2^ test.

3) To combine this within-gene information from (2) across all genes, a linear model was used to test the association between SNPs affecting the trait and SNPs affecting gene expression across the whole genome but on a within gene basis using model 5.

5 $$ \boldsymbol{y}=\mathbf{1}\upalpha +{\boldsymbol{X}}_{\mathbf{3}}{b}_3+{\boldsymbol{X}}_{\mathbf{4}}{b}_4+\boldsymbol{e} $$

**y** is an N × 1 vector, where y _*i*_ = 1 if the SNP was significantly associated with gene expression (*p* < 0.001) and 0 otherwise, and N is the number of SNPs. **1** is an N × 1 vector of 1’s and ɑ is a scalar intercept. **X**_**3**_ is an N × M design matrix where M = number of genes, allocating SNPs to their corresponding gene, and **b**_**3**_ is an M × 1 vector with b_3 i_ = the effect of gene *i* on gene expression. **X**_**4**_ is an N × 1 vector with X_4i_ = 1 if the SNP had a significant effect (*p* < 0.001) on the complex trait and 0 otherwise and b_4_ is a scalar measuring the association between GWAS and gene expression results. **e** is the vector of random residual effects ~ N(0, **I**σ^2^_e_).

A positive correlation between gene expression and GWAS results (b_4_ significantly positive) indicates that the QTL are more likely to be eQTL and its *p*-value shows how reliable the association is.

## Additional files

Additional file 1: **Table S1.** RNA-Seq data. The raw reads, QC passed and aligned paired reads and concordant pair alignment rate of the animals in each RNA-Seq dataset. (XLSX 27 kb)

Additional file 2: Contains supplementary materials including Figure S1 and Tables S2- S11. (DOCX 362 kb)

Additional file 3: **Table S12.** GWAS using SNP-chip data. The detailed results of *p*-values for each SNP in GWA studies for 20 traits and a multiple trait test combining these 20 traits. (CSV 258066 kb)

Additional file 4: **Table S13.** GWAS using imputed whole genome sequence data. The detailed results of p-values for each SNP in GWA study for residual feed intake. (CSV 674497 kb)

Additional file 5: **Table S14.** Overlap between eQTL and QTL. The detailed results of Χ^2^ tests for comparing QTL and eQTL to find whether SNP associated with conventional phenotypes (QTL) are also associated with gene expression in the local eQTL or ASE analyses (*p* < 0.05). (XLSX 114 kb)

Additional file 6: **Table S15.** Overlap between eQTL and QTL for each gene. The detailed results of Χ^2^ tests for comparing QTL and eQTL for each individual gene (*p* < 0.05). (XLSX 1381 kb)
