# Supplementary material for: Comparing allele specific expression and local expression quantitative trait loci and the influence of gene expression on complex trait variation in cattle
Source: BMC Genomics. 2018 Nov 3;19:793. doi: 10.1186/s12864-018-5181-0 (PMC6215656; doi:10.1186/s12864-018-5181-0)
Supplement: Supplementary file 2 — Contains supplementary materials including Figure S1 and Tables S2- S11. (DOCX 362 kb) [file 12864_2018_5181_MOESM2_ESM.docx]

**Table S2. Number of SNP in ASE and PO-ASE analyses.**

| **RNA-Seq data** | **No. of tSNP** | **No. of tSNP analysed** | **No. of tests**  **(tSNP-dSNP combination)** | **No. of unique dSNP** |
| --- | --- | --- | --- | --- |
| **Angus muscle** | 12,021 | 9,644 | 3,749,255 | 1,104,748 |
| **Angus liver** | 22,856 | 9,135 | 3,766,137 | 998,184 |
| **Holstein liver** | 28,243 | 20,822 | 8,639,073 | 2,029,634 |
| **Holstein WBC** | 35,481 | 26,418 | 11,419,065 | 2,366,448 |

The number of tSNP (SNP in the transcripts) and the number of tSNP used in ASE and PO-ASE analyses in each database (where at least 2 heterozygous animals received opposite alleles from their sire. Some of the dSNP (SNPs located within 50 kb of tSNP) were tested with multiple tSNP, so the number of unique dSNP is also provided.

**Table S3. Number of genes and SNP in eQTL mapping analyses.**

| **RNA-Seq data** | **No. of genes** | **No. of genes analysed** | **No. of tests**  **(gene-dSNP combination)** | **No. of unique dSNP** |
| --- | --- | --- | --- | --- |
| **Angus muscle** | 19,067 | 12,278 | 8,364,720 | 5,389,712 |
| **Angus liver** | 18,931 | 12,233 | 7,326,437 | 4,651,308 |
| **Holstein liver** | 18,828 | 14,373 | 9,866,261 | 5,962,727 |
| **Holstein WBC** | 18,782 | 14,175 | 9,641,386 | 5,592,577 |

Among the genes expressed in each RNA-Seq dataset, the genes expressed in more than 25% of samples were used in eQTL mapping. Some of the dSNP (SNPs located within 50 kb of the genes) were located within more than one gene region and were therefore tested multiple times, so the number of unique dSNP is also provided.

**Table S4. Comparisons between ASE discovered in different RNA-Seq databases (*p* < 0.001).**

| **ASE RNA-Seq1** | **ASE RNA-Seq2** | **No. of common tests** | **No. of significant tests in RNA-Seq1** | **No. of significant tests in RNA-Seq2** | **No. of significant tests in both** | **Fold enrichment** | **Same direction** |
| --- | --- | --- | --- | --- | --- | --- | --- |
| **Holstein WBC** | **Holstein Liver** | 4,152,056  (38,301,826) | 18,943  (163,503) | 10,989  (128,131) | 1,375  (3,769) | 27.43  (6.89) | 100%  (87%) |
| **Holstein WBC** | **Angus Liver** | 652,740  (7,506,092) | 2,884  (31,570) | 2,434  (27,390) | 1  (118) | 0.09  (1.02) | 100%  (100%) |
| **Holstein WBC** | **Angus Muscle** | 868,776  (9,149,445) | 3,228  (40,155) | 7,396  (110,917) | 85  (1,629) | 3.09  (3.35) | 100%  (77%) |
| **Holstein Liver** | **Angus Liver** | 1,143,578  (24,560,923) | 6,772  (150,789) | 8,794  (221,385) | 614  (11,500) | 11.79  (8.46) | 100%  (98%) |
| **Holstein Liver** | **Angus Muscle** | 1,024,462  (10,667,843) | 4,042  (55,065) | 8,745  (98,243) | 303  (1,725) | 8.78  (3.4) | 100%  (72%) |
| **Angus Liver** | **Angus Muscle** | 1,027,832  (7,731,213) | 4,389  (38,566) | 7,236  (77,917) | 1,076  (2,234) | 34.82  (5.75) | 100%  (99%) |
| **Average** |  | 1,478,241  (16,319,557) | 6,710  (79,941) | 7,599  (110,664) | 575.67  (3,496) | 14.33  (4.81) | 100 %  (89%) |

The SNP tested and significantly associated with allelic imbalance (*p* < 0.001) in pairs of databases. In the majority of tests fold enrichment was high and also the same allele is associated with higher expression in both cases (same direction). To compare the results for each gene, the tSNP and matching dSNP were selected, in parentheses are comparisons for the same dSNP but any tSNP within that gene.

**Table S5. Comparisons between PO-ASE discovered in different RNA-Seq databases (*p* < 0.001).**

| **PO-ASE RNA-Seq1** | **PO-ASE RNA-Seq2** | **No. of common tests** | **No. of significant tests in RNA-Seq1** | **No. of significant tests in RNA-Seq2** | **No. of significant tests in both** | **Fold enrichment** | **Same direction** |
| --- | --- | --- | --- | --- | --- | --- | --- |
| **Holstein WBC** | **Holstein Liver** | 41,52,056  (38,301,826) | 4,602  (35,294) | 3,363  (30,229) | 21  (152) | 5.63  (5.46) | 100%  (100%) |
| **Holstein WBC** | **Angus Liver** | 652,740  (7,506,092) | 646  (8,926) | 563  (7,342) | 0  (0) | 0  (0) | NA  (NA) |
| **Holstein WBC** | **Angus Muscle** | 868,776  (9,149,445) | 1,012  (6,463) | 2,343  (28,781) | 0  (165) | 0  (8.12) | NA  (100%) |
| **Holstein Liver** | **Angus Liver** | 1,143,578  (24,560,923) | 787  (13,716) | 1,090  (25,629) | 0  (51) | 0  (3.56) | NA  (4%) |
| **Holstein Liver** | **Angus Muscle** | 1,02,4462  (10,667,843) | 1,138  (7,993) | 2,169  (16,289) | 17  (92) | 7.06  (7.54) | 100%  (60%) |
| **Angus Liver** | **Angus Muscle** | 1,027,832  (7,731,213) | 1,512  (7,373) | 4,651  (38,557) | 189  (189) | 27.62  (5.14) | 100%  (100%) |
| **Average** |  | 1,478,241  (16,319,557) | 1,616  (13,294) | 2,363  (24,471) | 37.83  (108) | 6.72  (4.97) | 100 %  (66%) |

The SNP tested and significantly associated with allelic imbalance due to parent of origin of the alleles (*p* < 0.001) in different PO-ASE measurements were compared. To compare the results, for each gene the tSNP and matching dSNP were selected, but we also made some comparisons for the same dSNP but any tSNP within that gene, which are given in parentheses. Generally, comparing the results according to the same tSNP and same dSNP for each gene caused higher percentage of SNPs have same direction.

**Table S6. Comparisons between local eQTL discovered in different RNA-Seq databases (*p* < 0.001).**

| **local eQTL RNA-Seq1** | **Local eQTL RNA-Seq2** | **No. of common tests** | **No. of significant tests in RNA-Seq1** | **No. of significant tests in RNA-Seq2** | **No. of significant tests in both** | **Fold enrichment** | **Same direction** |
| --- | --- | --- | --- | --- | --- | --- | --- |
| **Holstein WBC** | **Holstein Liver** | 8,607,533 | 48,435 | 50,034 | 5,733 | 20.36 | 100% |
| **Holstein WBC** | **Angus Liver** | 5,269,793 | 33,264 | 45,112 | 1,709 | 6 | 99% |
| **Holstein WBC** | **Angus Muscle** | 5,794,740 | 32,726 | 72,795 | 4,458 | 10.84 | 96% |
| **Holstein Liver** | **Angus Liver** | 5,840,756 | 39,015 | 50,844 | 6,912 | 20.35 | 99% |
| **Holstein Liver** | **Angus Muscle** | 6,134,983 | 40,195 | 77,677 | 3,742 | 7.35 | 97% |
| **Angus Liver** | **Angus Muscle** | 6,093,564 | 44,763 | 74,570 | 6,726 | 12.28 | 94% |
| **Average** |  | 6,290,228 | 39,733 | 61,839 | 4,880 | 12.87 | 97% |

The SNP tested and significantly associated with gene expression (*p* < 0.001) in different local eQTL mapping datasets. The matching dSNP within each gene were selected to compare the results.

**Table S7. Comparisons between ASE and eQTL discovered in different RNA-Seq databases (*p* < 0.001).**

| **ASE**  **RNA-Seq1** | **eQTL mapping RNA-Seq2** | **No. of common tests** | **No. of significant tests in RNA-Seq1** | **No. of significant tests in RNA-Seq2** | **No. of significant tests in both** | **Fold enrichment** | **Same direction** |
| --- | --- | --- | --- | --- | --- | --- | --- |
| **Holstein WBC** | **Holstein WBC** | 11,405,529 | 45,312 | 93,827 | 4,558 | 12.23 | 87% |
| **Holstein WBC** | **Holstein Liver** | 11,300,111 | 44,028 | 167,077 | 5,525 | 8.49 | 64% |
| **Holstein WBC** | **Angus Liver** | 8,410,962 | 37,805 | 99,462 | 941 | 2.1 | 61% |
| **Holstein WBC** | **Angus Muscle** | 8,623,032 | 35,913 | 175,499 | 2,720 | 3.72 | 82% |
| **Holstein Liver** | **Holstein WBC** | 7,922,422 | 25,826 | 77,749 | 220 | 0.87 | 62% |
| **Holstein Liver** | **Holstein Liver** | 8,628,027 | 27,417 | 138,592 | 5,293 | 12.02 | 97% |
| **Holstein Liver** | **Angus Liver** | 6,800,216 | 24,885 | 136,398 | 3,893 | 7.8 | 99% |
| **Holstein Liver** | **Angus Muscle** | 6,495,814 | 22,990 | 150,831 | 1,008 | 1.89 | 62% |
| **Angus Liver** | **Holstein WBC** | 2,694,076 | 15,524 | 31,232 | 322 | 1.79 | 100% |
| **Angus Liver** | **Holstein Liver** | 3,202,576 | 22,132 | 88,937 | 3,283 | 5.34 | 95% |
| **Angus Liver** | **Angus Liver** | 3,753,829 | 21,463 | 100,167 | 6,841 | 11.94 | 98% |
| **Angus Liver** | **Angus Muscle** | 2,927,419 | 14,298 | 87,838 | 729 | 1.7 | 92% |
| **Angus Muscle** | **Holstein WBC** | 2,757,090 | 21,329 | 28,617 | 443 | 2 | 91% |
| **Angus Muscle** | **Holstein Liver** | 2,923,165 | 20,152 | 38,276 | 2,573 | 9.75 | 100% |
| **Angus Muscle** | **Angus Liver** | 3,079,782 | 22,402 | 27,792 | 812 | 4.02 | 100% |
| **Angus Muscle** | **Angus Muscle** | 3,745,073 | 24,240 | 96,540 | 3,679 | 5.89 | 98% |
| **Average** |  | 5,916,820 | 26,607 | 96,177 | 2,678 | 5.72 | 87% |

The SNP tested and significantly associated with allelic imbalance in one dataset and local eQTL mapping in another (p < 0.001). According to the results, the fold enrichments are increased in comparisons within datasets which indicates ASE and local eQTL both detect the same effects.

**Table S8. Validation of discovered ASE in reported ASE in 18 tissues (*p* < 0.01).**

|  | **WBC** | | | **Liver (Holstein)** | | | **Liver (Angus)** | | | **Muscle** | | | **Average** | | |
| --- | --- | --- | --- | --- | --- | --- | --- | --- | --- | --- | --- | --- | --- | --- | --- |
| **Validation tissue** | **No. of test** | **No. of Sig. test** | **Same direction** | **No. of test** | **No. of Sig. test** | **Same direction** | **No. of test** | **No. of Sig. test** | **Same direction** | **No. of test** | **No. of Sig. test** | **Same direction** | **No. of test** | **No. of Sig. test** | **Same direction** |
| **Adrenal gland** | 5,750 | 64*** | 94% | 4,881 | 62*** | 92% | 1,574 | 43*** | 81% | 1,811 | 42*** | 86% | 3,045.00 | 52.75 | 88.20% |
| **Black skin** | 5,982 | 89*** | 85% | 5,073 | 84*** | 79% | 1,626 | 28 | 64% | 1,846 | 46*** | 83% | 3,151.33 | 61.75 | 77.71% |
| **WBC** | 6,285 | 92*** | 92% | 4,331 | 54*** | 83% | 1,343 | 25*** | 76% | 1,589 | 40*** | 88% | 3,072.33 | 52.75 | 84.81% |
| **Brain caudal lobe** | 6,400 | 76*** | 95% | 4,943 | 49*** | 96% | 1,551 | 24*** | 92% | 1,835 | 35*** | 89% | 3,262.00 | 46.00 | 92.72% |
| **Brain cerebellum** | 5,669 | 61*** | 92% | 4,738 | 46*** | 93% | 1,521 | 20** | 85% | 1,821 | 41*** | 85% | 3,003.67 | 42.00 | 88.91% |
| **Heart** | 3,901 | 62** | 69% | 3,819 | 65*** | 88% | 1,347 | 25 | 76% | 1,824 | 44** | 89% | 2,357.33 | 49.00 | 80.42% |
| **Kidney** | 6,014 | 112 | 76% | 5,303 | 110* | 76% | 1,734 | 57 | 77% | 1,851 | 65*** | 72% | 3,199.67 | 86.00 | 75.44% |
| **Leg muscle** | 4,795 | 44** | 82% | 4,301 | 43*** | 95% | 1,386 | 29*** | 79% | 2,036 | 42*** | 98% | 2,739.00 | 39.50 | 88.52% |
| **Liver** | 4,788 | 124** | 67% | 4,957 | 127*** | 82% | 1,890 | 78 | 76% | 1,697 | 63** | 62% | 2,791.67 | 98.00 | 71.59% |
| **Lung** | 5,399 | 140 | 71% | 4,681 | 122 | 71% | 1,575 | 57 | 61% | 1,771 | 74** | 65% | 2,915.00 | 98.25 | 67.07% |
| **Intestinal lymph node** | 5,204 | 79 | 80% | 4,164 | 54 | 81% | 1,394 | 28 | 86% | 1,600 | 45*** | 78% | 2,732.67 | 51.50 | 81.18% |
| **Mammary gland** | 3,461 | 29 | 83% | 3,643 | 41*** | 73% | 1,316 | 16 | 81% | 1,546 | 35*** | 83% | 2,107.67 | 30.25 | 80.01% |
| **Ovary** | 5,849 | 66*** | 94% | 4,898 | 58*** | 93% | 1,566 | 25*** | 80% | 1,814 | 36*** | 92% | 3,076.33 | 46.25 | 89.68% |
| **Spleen** | 6,189 | 60*** | 97% | 4,811 | 49*** | 94% | 1,508 | 28*** | 86% | 1,753 | 38*** | 89% | 3,150.00 | 43.75 | 91.43% |
| **Thymus** | 4,549 | 33*** | 91% | 3,590 | 31*** | 84% | 1,239 | 11** | 64% | 1,485 | 19*** | 100% | 2,424.33 | 23.50 | 84.60% |
| **Thyroid gland** | 6,314 | 95*** | 93% | 5,272 | 85*** | 86% | 1,667 | 45*** | 80% | 1,900 | 47*** | 83% | 3,293.67 | 68.00 | 85.37% |
| **Tongue** | 5,071 | 54*** | 89% | 4,591 | 49*** | 96% | 1,483 | 26*** | 81% | 2,040 | 47*** | 81% | 2,864.67 | 44.00 | 86.61% |
| **White skin** | 6,202 | 84*** | 89% | 5,224 | 70*** | 90% | 1,690 | 33*** | 85% | 1,873 | 42*** | 81% | 3,255.00 | 57.25 | 86.27% |
| **Average** | 5,434.56 | 75.78 | 85.42% | 4,623.33 | 66.61 | 86.29% | 1522.78 | 33.22 | 78.32% | 1,782.89 | 44.50 | 83.43% | 2,913.41 | 55.03 | 83.36% |

The SNP significantly associated with allelic imbalance (*p* < 0.01) in validation tissues and the 4 datasets of this study. The majority of chi-squared tests were significant (*, ** and *** show significance level 0.05, 0.01 and 0.001, respectively) indicating similar ASE were found in our study.

**Table S9. Validation of local eQTL in reported ASE in 18 tissues (*p* < 0.01).**

|  | **Holstein WBC** | | | **Holstein Liver** | | | **Angus Liver** | | | **Angus Muscle** | | | **Average** | | |
| --- | --- | --- | --- | --- | --- | --- | --- | --- | --- | --- | --- | --- | --- | --- | --- |
| **Validation tissue** | **No. of test** | **No. of Sig. test** | **Same direction** | **No. of test** | **No. of Sig. test** | **Same direction** | **No. of test** | **No. of Sig. test** | **Same direction** | **No. of test** | **No. of Sig. test** | **Same direction** | **No. of test** | **No. of Sig. test** | **Same direction** |
| **Adrenal gland** | 13,366 | 163*** | 74% | 13,905 | 164*** | 71% | 11,351 | 114*** | 74% | 12,319 | 225*** | 70% | 12,345 | 167 | 72% |
| **Black skin** | 14,375 | 206** | 64% | 15,125 | 206 | 56% | 12,115 | 138 | 53% | 13,118 | 277*** | 65% | 13,203 | 207 | 59% |
| **WBC** | 12,478 | 211*** | 76% | 12,345 | 170** | 67% | 9,990 | 106 | 62% | 10,264 | 200*** | 67% | 10,911 | 172 | 68% |
| **Brain caudal lobe** | 14,780 | 156*** | 78% | 15,197 | 142** | 68% | 12,059 | 94* | 65% | 12,796 | 180*** | 73% | 13,212 | 143 | 71% |
| **Brain cerebellum** | 13,625 | 119** | 68% | 14,133 | 108 | 65% | 11,236 | 83 | 73% | 12,428 | 159*** | 73% | 12,430 | 117 | 70% |
| **Heart** | 8,743 | 117 | 73% | 9,073 | 169*** | 60% | 7,467 | 92 | 61% | 8,400 | 211* | 67% | 8,203 | 147 | 65% |
| **Kidney** | 14,709 | 298 | 68% | 15,728 | 431*** | 67% | 12,797 | 245 | 67% | 13,370 | 398* | 62% | 13,625 | 343 | 66% |
| **Leg muscle** | 10,420 | 100** | 70% | 10,690 | 111* | 60% | 8,836 | 76 | 72% | 10,146 | 204*** | 76% | 9,801 | 123 | 70% |
| **Liver** | 11,296 | 260 | 64% | 12,178 | 409* | 69% | 10,258 | 302* | 66% | 10,074 | 376 | 58% | 10,543 | 337 | 64% |
| **Lung** | 12,852 | 388 | 59% | 13,533 | 454 | 53% | 10,978 | 308 | 57% | 11,481 | 464 | 54% | 11,770 | 404 | 56% |
| **Intestinal lymph node** | 10,937 | 170* | 66% | 11,164 | 189 | 55% | 9,172 | 127 | 60% | 9,595 | 252*** | 63% | 9,901 | 185 | 61% |
| **Mammary gland** | 7,448 | 78* | 77% | 7,742 | 61 | 64% | 6,456 | 69* | 70% | 6,895 | 109* | 73% | 6,933 | 79 | 71% |
| **Ovary** | 13,541 | 119*** | 76% | 14,240 | 127* | 63% | 11,515 | 85 | 73% | 12,568 | 182*** | 75% | 12,541 | 128 | 72% |
| **Spleen** | 13,450 | 137*** | 79% | 13,840 | 134* | 64% | 11,268 | 91 | 71% | 11,747 | 170*** | 68% | 12,155 | 133 | 71% |
| **Thymus** | 9,338 | 67*** | 76% | 9,352 | 76*** | 53% | 7,674 | 45 | 78% | 8,106 | 83** | 82% | 8,373 | 68 | 72% |
| **Thyroid gland** | 16,154 | 193*** | 72% | 17,086 | 237*** | 76% | 13,587 | 166*** | 72% | 14,627 | 284*** | 68% | 14,789 | 220 | 72% |
| **Tongue** | 11,561 | 90** | 79% | 11,973 | 110** | 65% | 9,830 | 67 | 73% | 11,221 | 172*** | 75% | 10,871 | 110 | 73% |
| **White skin** | 15,101 | 142 | 70% | 15,970 | 208*** | 75% | 12,766 | 130 | 69% | 13,704 | 243*** | 66% | 13,857 | 181 | 70% |
| **Average** | 12,454 | 167 | 72% | 12,960 | 195 | 64% | 10520 | 130 | 68% | 11,270 | 233 | 69% | 11,415 | 181 | 68% |

The SNP significantly associated with allelic imbalance (*p* < 0.01) in validation tissues and also in local eQTL mapping results from 4 datasets. The majority of chi-squared tests were significant (*, ** and *** show significance level 0.01, 0.05 and 0.001, respectively) indicating similar SNP were found as local eQTL in our study.

**Table S10. The names and abbreviations of the traits used in GWAS.**

| **Trait** | **Abbreviation** |
| --- | --- |
| Intramuscular percent fat measured in Longissimus lumborum muscle | CIMF |
| Australian meat marble score assessed by grader | CMARB |
| P8 (rump) fat depth at slaughter | CP8 |
| Carcass retail beef yield | CRBY |
| Rib fat at slaughter | CRIB |
| Blood concentration of Insulin-like Growth Factor I (IGF-I) measured at feedlot entry | E_IGF |
| Average daily gain over RFI test period | FI_ADG |
| Metabolic mid-test weight in the RFI test period | FI_MIDWT |
| Net feed intake | FI_NFI |
| Tenderness | MQLDPF |
| Scanned eye muscle area at slaughter | M_SEMA |
| Hip height measured post weaning | PW_HIP |
| Live weight measured post weaning | PW_LWT |
| IGF-I concentration in blood measured post weaning | PW_IGF |
| Hip height measured at feedlot entry | E_HIP |
| Hip height measured at feedlot exit | X_HIP |
| Live weight measured at feedlot exit | X_LWT |
| Exit scanned eye muscle area | X_SEMA |
| Exit scanned P8 fat depth | X_SP8 |
| Exit scanned rib fat | X_SRIB |
| Multi trait GWAS test | Multi-Trait |
| Residual feed intake | RFI |

The GWA studies for the traits given in the table, include 20 traits and 1 multi-trait, were based on 800K SNP chip genotypes. A second GWAS for RFI was based on WGS SNP.

**Table S11. Number of SNP tested and significantly associated with the traits (*p* < 0.001) in GWAS.**

|  | **Genome-wide** | | **Expressed in Angus muscle** | | **Expressed in Angus liver** | | **Expressed in Holstein liver** | | **Expressed in Holstein blood** | |
| --- | --- | --- | --- | --- | --- | --- | --- | --- | --- | --- |
| **Trait** | **No. of SNPs** | **No. of sig.** | **No. of SNPs** | **No. of sig.** | **No. of SNPs** | **No. of sig.** | **No. of SNPs** | **No. of sig.** | **No. of SNPs** | **No. of sig.** |
| **CIMF** | 663,169 | 1,132 | 237,756 | 409 | 225,863 | 362 | 260,415 | 438 | 240,269 | 406 |
| **CMARB** | 653,629 | 723 | 237,643 | 262 | 225,842 | 255 | 260,047 | 297 | 239,945 | 293 |
| **CP8** | 663,106 | 1,131 | 237,757 | 368 | 225,867 | 349 | 260,413 | 374 | 240,267 | 352 |
| **CRBY** | 658,928 | 1,195 | 237,724 | 398 | 225,859 | 377 | 260,271 | 417 | 240,136 | 367 |
| **CRIB** | 660,864 | 2,110 | 237,745 | 696 | 225,862 | 703 | 260,349 | 800 | 240,207 | 687 |
| **E_IGF** | 643,933 | 1,391 | 237,434 | 385 | 225,835 | 365 | 258,981 | 491 | 238,939 | 456 |
| **FI_ADG** | 650,047 | 919 | 237,630 | 313 | 225,841 | 293 | 259,891 | 352 | 239,792 | 324 |
| **FI_MIDWT** | 650,047 | 1,358 | 237,630 | 512 | 225,841 | 506 | 259,891 | 574 | 239,792 | 570 |
| **FI_NFI** | 656,759 | 2,652 | 237,712 | 1,006 | 225,865 | 969 | 260,216 | 980 | 240,100 | 957 |
| **MQLDPF** | 659,757 | 1,350 | 237,725 | 557 | 225,860 | 539 | 260,293 | 648 | 240,157 | 569 |
| **M_SEMA** | 625,138 | 1,085 | 232,882 | 442 | 221,784 | 436 | 254,839 | 488 | 235,065 | 473 |
| **PW_HIP** | 657,601 | 1,750 | 237,698 | 649 | 225,858 | 625 | 260,166 | 761 | 240,046 | 720 |
| **PW_LWT** | 663,464 | 1,624 | 237,768 | 576 | 225,867 | 528 | 260,409 | 612 | 240,280 | 588 |
| **PW_IGF** | 635,927 | 851 | 236,990 | 342 | 225,810 | 350 | 257,773 | 399 | 237,840 | 391 |
| **E_HIP** | 647,063 | 1,448 | 237,476 | 571 | 225,782 | 556 | 259,690 | 671 | 239,594 | 683 |
| **X_HIP** | 645,057 | 1,725 | 237,432 | 391 | 225,741 | 363 | 259,582 | 511 | 239,502 | 440 |
| **X_LWT** | 663,149 | 2,131 | 237,762 | 639 | 225,866 | 613 | 260,388 | 742 | 240,259 | 701 |
| **X_SEMA** | 662,974 | 1,105 | 237,764 | 305 | 225,866 | 326 | 260,406 | 392 | 240,280 | 356 |
| **X_SP8** | 663,532 | 1,793 | 237,767 | 624 | 225,867 | 599 | 260,416 | 717 | 240,290 | 624 |
| **X_SRIB** | 663,532 | 1,700 | 237,767 | 580 | 225,867 | 560 | 260,416 | 643 | 240,290 | 575 |
| **Multi-Trait** | 692,527 | 17,393 | 236,743 | 5,116 | 224,981 | 4,874 | 259,618 | 5,643 | 239,458 | 4,890 |
| **RFI** | 24,041,262 | 61,344 | 5,324,643 | 15,659 | 4,646,079 | 14,206 | 5,925,599 | 15,905 | 5,557,922 | 15,103 |

Number of SNP which were tested for each trait and the number of SNP located within 50kb of the genes expressed in Angus muscle and liver and Holstein liver and WBC samples, followed by the number of SNPs significantly associated with the traits (*p* < 0.001). In the GWAS for RFI, the SNP from WGS (real or imputed) were used and for the rest of the GWAS, the SNP were 800K SNP chip (real or imputed) genotypes.


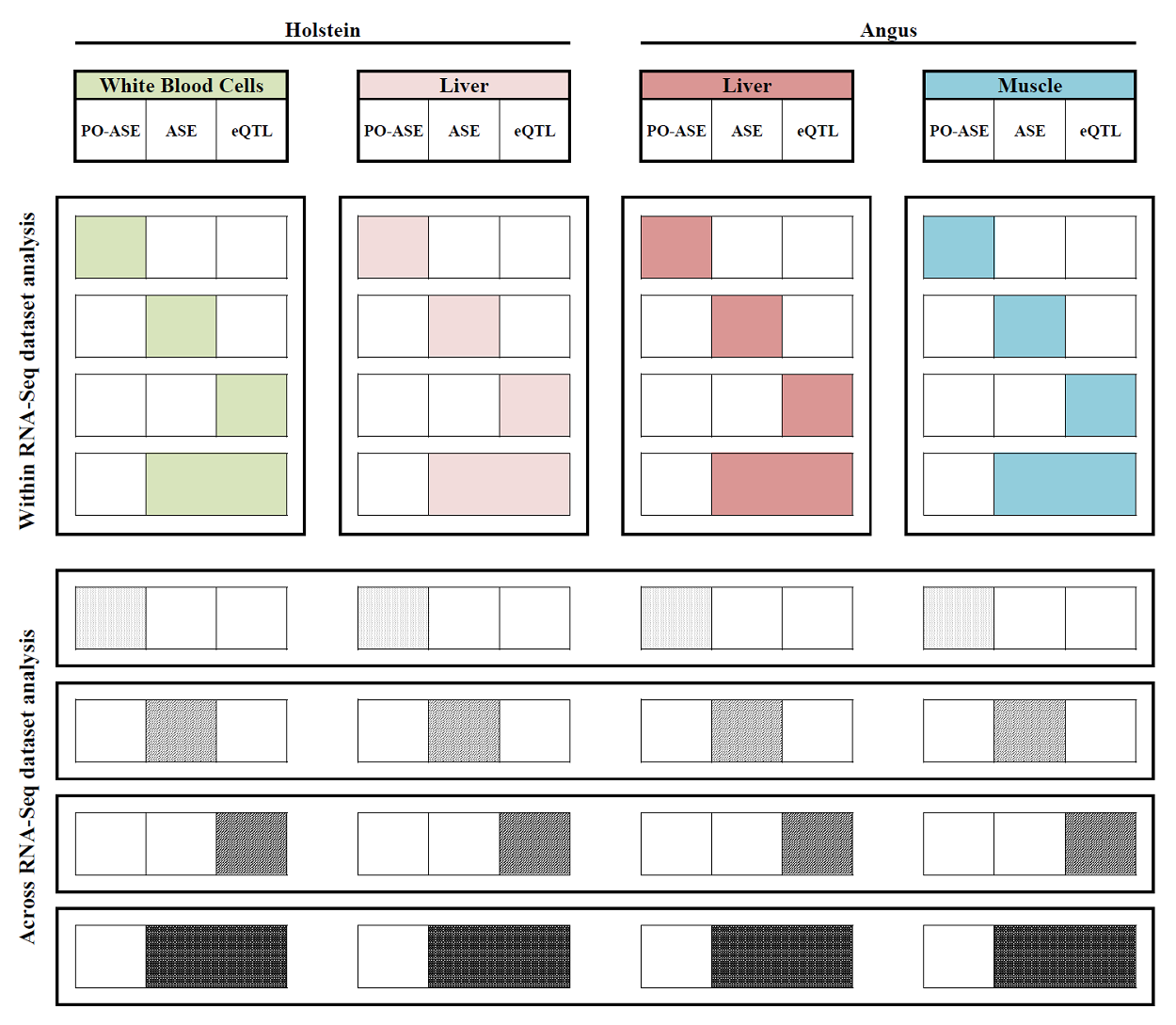


**Fig S1. The within and across RNA-Seq database analyses pattern.**

The within RNA-Seq database analyses are shown in 4 top boxes. In each database ASE, PO-ASE and eQTL analyses were performed independently and ASE and eQTL results were also combined within each database. The across database analyses are shown in 4 bottom boxes, ASE, PO-ASE, eQTL and ASE/eQTL are each combined across all 4 RNA-Seq databases.
